# Tuning Electrocatalytic Energy Release in Norbornadiene Based Molecular Solar Thermal Systems Through Substituent Effects

**DOI:** 10.1002/chem.202502294

**Published:** 2025-08-06

**Authors:** Evanie Franz, Nils Oberhof, Daniel Krappmann, Nicolò Baggi, Zarah Hussain, Kasper Moth‐Poulsen, Helen Hölzel, Andreas Hirsch, Andreas Dreuw, Olaf Brummel, Jörg Libuda

**Affiliations:** ^1^ Interface Research and Catalysis Erlangen Center for Interface Research and Catalysis Friedrich‐Alexander‐Universität Erlangen‐Nürnberg Egerlandstraße 3 Erlangen 91058 Germany; ^2^ Interdisciplinary Center for Scientific Computing Universität Heidelberg Im Neuenheimer Feld 205 A Heidelberg 69120 Germany; ^3^ Chair of Organic Chemistry II Friedrich‐Alexander‐Universität Erlangen‐Nürnberg Nikolaus‐Fiebiger‐Straße 10 Erlangen 91058 Germany; ^4^ The Institute of Materials Science of Barcelona ICMAB‐CSIC Ballaterra Barcelona 08193 Spain; ^5^ Department of Chemical Engineering Universitat Politècnica de Catalunya EEBE, Eduard Maristany 10–14 Barcelona 08019 Spain; ^6^ Catalan Institution for Research & Advanced Studies ICREA Pg. Lluís Companys 23 Barcelona 08010 Spain; ^7^ Department of Chemistry and Chemical Engineering Chalmers University of Technology Gothenburg SE‐412 96 Sweden; ^8^ Institut Organische Chemie Justus‐Liebig‐Universität Gießen Heinrich‐Buff‐Ring 17 Gießen 35392 Germany

**Keywords:** electrochemistry, IR spectroscopy, isomerization, photoswitches, push–pull system

## Abstract

Molecular solar thermal (MOST) systems, such as the norbornadiene/quadricyclane (NBD/QC) pair, combine solar energy conversion, storage, and release in a simple one‐molecule process. The energy‐releasing reaction QC to NBD can be controlled electrochemically. In this study, we used in‐situ photoelectrochemical infrared spectroscopy (PEC‐IRRAS) together with density functional theory (DFT) calculations to investigate how electron donating (EDG) and electron withdrawing (EWG) groups in the push‐pull system of the MOST pair affect the electrocatalytic properties of the electrochemically triggered back‐conversion. Specifically, we investigated cyano, tosyl, and methyl ester groups as EWGs, and methoxy, dimethylamine, thioether, and diphenylamine groups located in the *para*‐position of a phenyl group as EDGs. We characterized the onset potential, electrochemical stability window, and selectivity. We found that these properties strongly depend on the strength of electron donation of the EDG, as it exclusively locates the highest occupied molecular orbital (HOMO) and raises its energy level. We obtained the highest selectivity for compounds with *p*‐methoxyphenyl functionality.

## Graphical abstract

We investigated how push–pull substituents influence the electrochemically driven energy release in norbornadiene‐based MOST systems. Electron‐donating groups proved to be decisive, with p‐methoxyphenyl derivatives achieving up to 99% selectivity. These insights offer valuable design guidelines for future MOST systems.

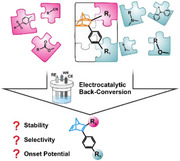

## 1 Introduction

**Figure 1:**
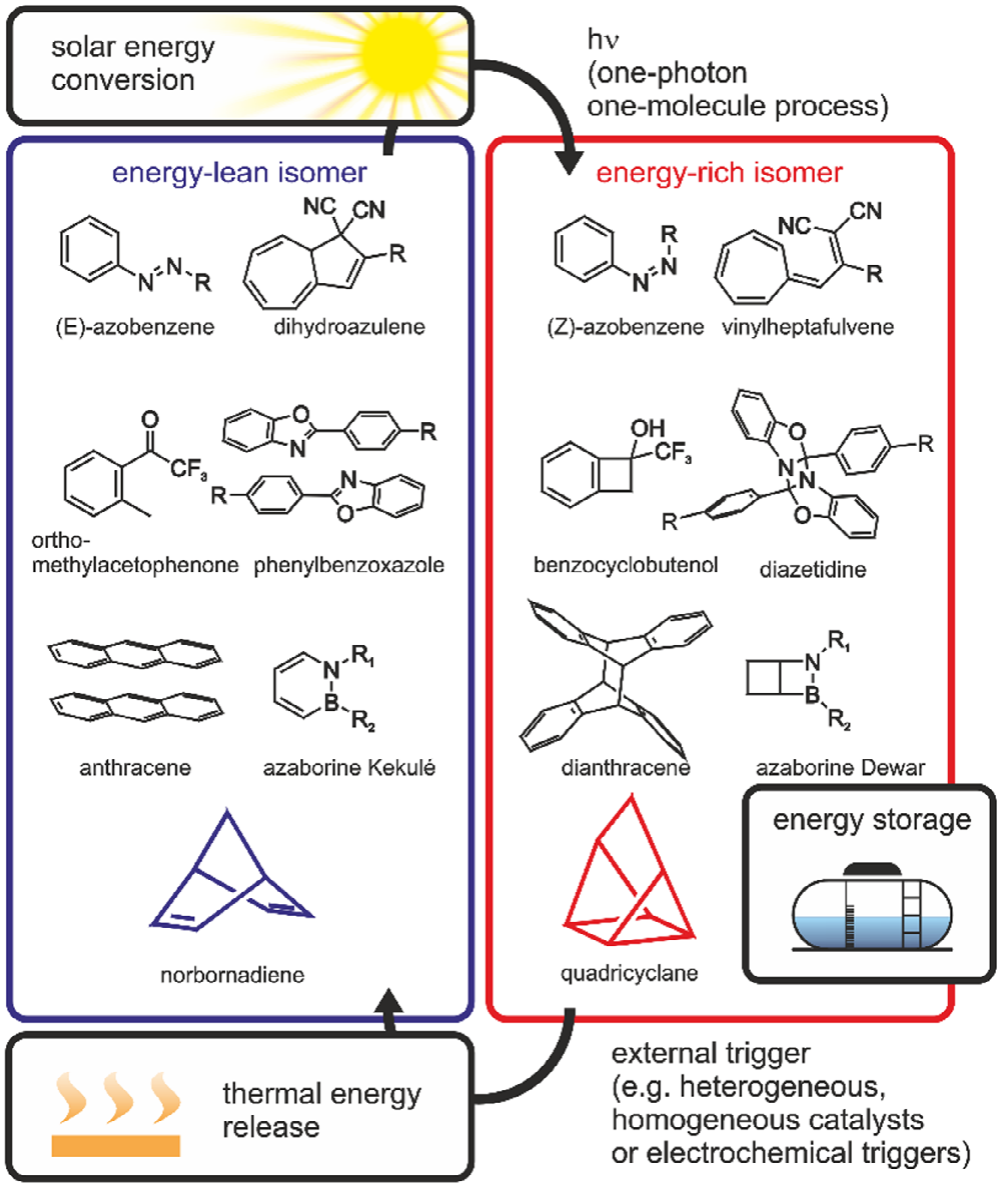
Conceptual overview of various MOST systems, illustrating the integration of solar energy conversion, energy storage, and controlled energy release within a single molecular framework.

In recent years, the field of molecular solar thermals (MOSTs) has gained increasing interest as a means of storing solar energy.^[^ ^1^, ^2^ ^]^ They consist of so‐called photoswitches, molecules that undergo an isomerization reaction upon absorption of light of a certain wavelength (Figure 1). They convert from a low‐energy isomer to a high‐energy metastable isomer, thus storing the absorbed energy.^[^ ^3^ ^]^ There are several examples of compounds that can undergo this reaction, such as the (*E*)/(*Z*)‐azobenzene pair,^[^ ^4^, ^5^, ^6^ ^]^ the dihydroazulene/ vinylheptafulvene pair,^[^ ^7^ ^]^ the ortho‐methylacetophenone/benzocyclobutenol pair,^[^ ^8^ ^]^ the phenylbenzoxazole/diazetidine pair,^[^ ^9^ ^]^ the anthracene/dianthracene pair,^[^ ^10^ ^]^ and the azaborine Kekulé‐/Dewar conformer pair.^[^ ^11^ ^]^ A particularly well‐developed MOST system is the norbornadiene/quadricyclane (NBD/QC) pair, in which the NBD undergoes a [2 + 2] cycloaddition upon exposure to UV light to form QC.^[^ ^12^, ^13^, ^14^, ^15^ ^]^

To be suitable for practical applications, MOST systems must fulfill a range of key requirements. These include: (1) the molecular absorption range, (2) the photoisomerization quantum yield (ϕ_iso_), (3) the energy storage time, (4) the energy storage density (ΔH_storage_), (5) the absorption spectrum difference between parent and photoisomer states, (6) environmental impact and toxicity, (7) the triggering mechanism for energy release, and (8) the cyclability. In the following, we briefly discuss these critical criteria. For a more detailed discussion, we refer to our review article.^[^ ^1^ ^]^

1. Since more than 50% of solar photons reaching Earth's surface lie within the 300–800 nm range, an ideal MOST molecule should exhibit strong absorption across a substantial portion of this spectrum to efficiently harvest sunlight.^[^ ^16^ ^]^
2. The photoisomerization quantum yield (ϕ_iso_), which quantifies how efficiently absorbed photons induce isomerization, should be as close to unity as possible to maximize the utilization of incoming light.^[^ ^1^ ^]^
3. The energy storage time is directly linked to the activation enthalpy barrier (ΔH^‡^). To ensure practical energy retention, the thermal half‐life (t_1_/_2_) at room temperature should allow storage over days (ΔH^‡^ ≥ 110 kJ mol^−1^), months (ΔH^‡^ ≥ 120 kJ mol^−1^), or even years (ΔH^‡^ ≥ 130 kJ mol^−1^), depending on the intended application.^[^ ^16^, ^17^ ^]^
4. The energy storage density (ΔH_storage_) of MOST systems should exceed that of conventional materials such as salt hydrates and ideally reach at least 0.3 MJ kg^−1^.^[^ ^18^, ^19^ ^]^
5. The absorption spectra of the parent and photoisomer states should be well‐separated to prevent spectral overlap, thereby avoiding competition between light‐induced forward and backward isomerization processes.^[^ ^1^ ^]^
6. MOST compounds should be environmentally benign and nontoxic to support their safe use in large‐scale and long‐term applications.^[^ ^1^ ^]^
7. The trigger used to revert the metastable isomer to its original state must enable controlled, selective, and reliable energy release on demand.^[^ ^20^ ^]^
8. Selectivity in both the photochemical conversion and the triggered back‐conversion is essential, as it directly affects cyclability. High cyclability ensures that the MOST system can undergo numerous charge–discharge cycles without molecular fatigue or irreversible degradation, thereby maintaining consistent long‐term performance.^[^ ^20^, ^21^ ^]^

In the past, research has focused primarily on the photochemical and energy storage properties. For example, in the case of the NBD/QC system investigated in this work, unsubstituted NBD absorbs light only at wavelengths below 300 nm, necessitating the use of additional photosensitizers to capture sunlight.^[^ ^18^, ^22^, ^23^ ^]^ To shift the absorption maximum of the NBD to the desired region (see requirement 1), so‐called push‐pull systems are employed. These consist of electron‐donating groups (EDG) and electron‐withdrawing groups (EWG) typically attached at the 2‐ and 3‐positions, lowering the HOMO‐LUMO gap and thus red‐shifting the absorption.^[^ ^24^, ^25^, ^26^ ^]^ This approach has resulted in a large library of NBD/QC derivatives with different absorption spectra, half‐lives, and energy densities,^[^ ^24^, ^25^, ^26^, ^27^, ^28^, ^29^ ^]^ addressing multiple aspects of the aforementioned requirements.

Initially, much less attention was paid to the controlled release of energy. In recent years, however, the development of suitable triggers to initiate the energy release has come into increasing focus.^[^ ^21^, ^30^, ^31^, ^32^ ^]^ Various concepts have been reported in the literature, including light,^[^ ^2^, ^33^, ^34^ ^]^ heat,^[^ ^25^, ^26^, ^35^ ^]^ mechanochemistry,^[^ ^36^ ^]^ homogeneous,^[^ ^37^ ^]^ quasi‐homogeneous^[^ ^24^ ^]^ or heterogeneous catalysis.^[^ ^21^, ^30^, ^31^, ^38^, ^39^, ^40^ ^]^ Another promising approach is to trigger the energy release electrochemically.^[^ ^41^ ^]^ This approach requires hardly any additional energy input^[^ ^42^ ^]^ due to the proposed autocatalytic reaction mechanism and the overall nonredox nature of the reaction.^[^ ^42^, ^43^ ^]^ Further, this approach enables straightforward MOST trigger separation, and reaches high reversibility of up to 99.8%.^[^ ^42^, ^44^ ^]^ Finally, the approach has the advantage that the reaction kinetics can be easily controlled by the applied potential.^[^ ^41^, ^44^ ^]^

In a recent study, we demonstrated that the push‐pull system introduced to tune the photochemical properties of the MOST system does not only influence the absorption maximum of the NBD, but also significantly influences the electrochemically triggered energy release. Specifically, the presence of a single methoxy group on the EDG increased the back‐conversion selectivity from 60% to > 99%.^[^ ^20^, ^42^ ^]^ However, that study was limited to only two NBD derivatives and therefore did not allow the derivation of general structure–reactivity relationships.

In the present work, we address this limitation by conducting the first systematic investigation of how different push–pull substituents influence the electrochemical behavior of NBD/QC systems. By combining spectroelectrochemical experiments with established DFT‐based analyses, we examine the impact of a broad range of electron‐donating substituents on key properties, including onset potential, electrochemical stability, and back‐conversion selectivity. The novelty of this study lies in the identification of consistent structure–property trends, enabling the formulation of general design principles for MOST systems with electrochemically triggered energy release. These insights offer a deeper mechanistic understanding of substituent effects and provide a valuable foundation for the rational development of future MOST materials.

## 2 Results and Discussion

**Figure 2:**
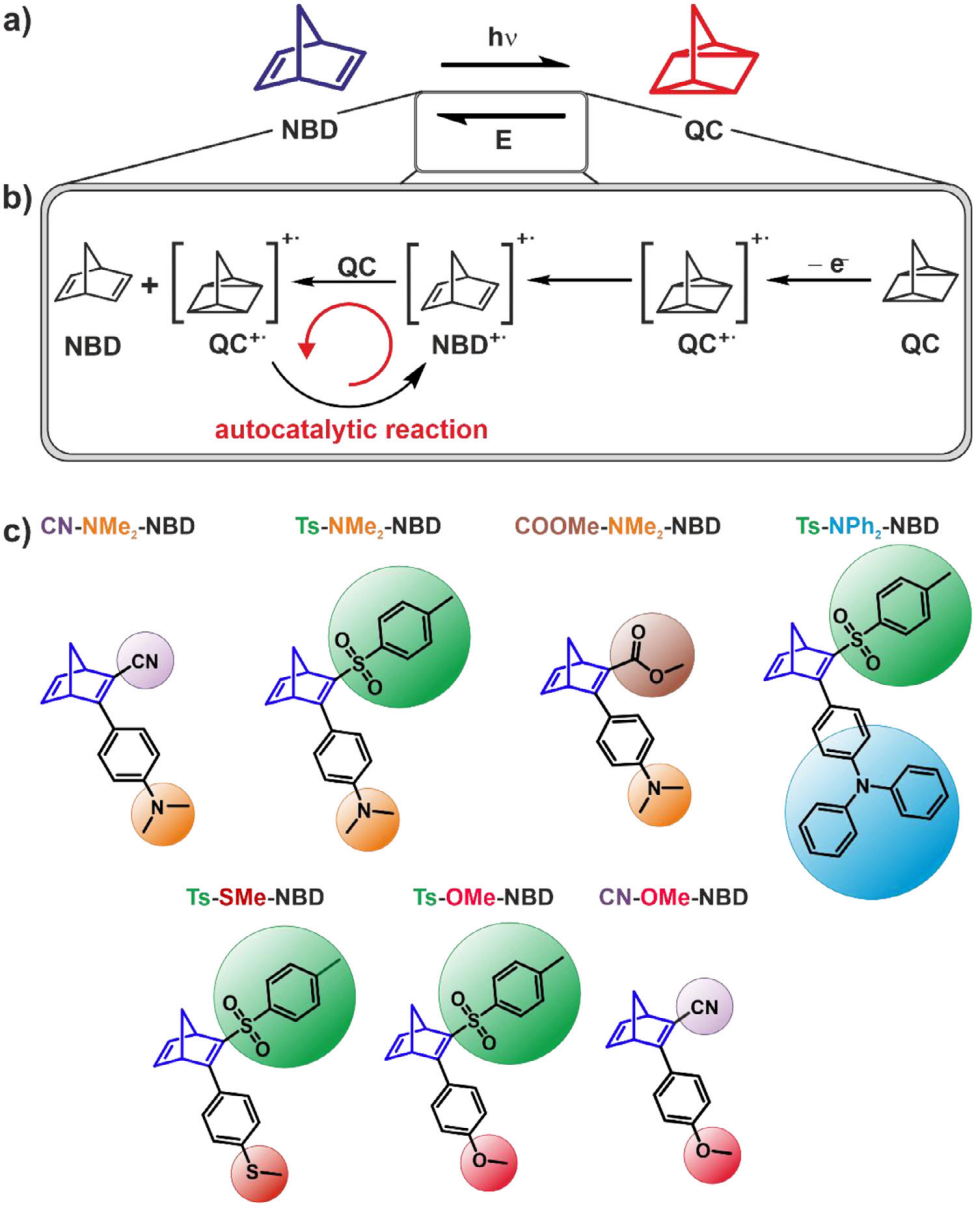
Overview of the electrochemically triggered back‐conversion. a) Conversion and electrocatalytic back‐conversion of NBD to QC; b) reaction mechanism of the electrocatalytically triggered back‐conversion reported in literature^[^ ^43^ ^]^; c) NBD derivatives investigated in this study.

In Figure 2a, b, we schematically depict the reaction mechanism of the electrochemically triggered back‐conversion as proposed in literature.^[^ ^1^, ^41^, ^43^ ^]^ The oxidation of QC generates QC^+•^,^·^which reacts to NBD^+•^. This initiates a radical chain reaction that drives the back‐conversion of QC to NBD via an autocatalytic mechanism. In this work, we have investigated substituents with different push‐pull functionalities by electrochemically triggered back‐conversion. In particular, we investigate the onset potential, the electrochemical stability window, and the selectivity. As EWG we used cyano (‐CN), methyl ester (‐COOMe), and tosyl (Ts) groups. As EDG, we used phenyl groups with a dimethylamino (NMe_2_), a diphenylamino (NPh_2_), a thioether (‐SMe), or a methoxy (‐OMe) group located always in *para*‐position. The investigated compounds are shown in Figure 2c, and the most important physicochemical properties for MOST applications are summarized in Table 1.

**Table 1:** Summary of the physicochemical properties for MOST compounds studied in this paper.^[^ ^26^, ^45^ ^]^

Compound	CN‐NMe_2_‐NBD/QC	Ts‐NMe_2_‐NBD/QC	COOMe‐NMe_2_‐NBD/QC	Ts‐NPh_2_‐NBD/QC	Ts‐SMe‐NBD/QC	Ts‐OMe‐NBD/QC	CN‐OMe‐NBD/QC
λ_max_ measured [nm]	373	367	366	370	324	312	326
t_1/2_ [days]	7	38	103	115	4	4	30
Calculated ΔH_storage_ [kJ mol^−1^]	110.8	63.8	91.8	80.9	60.6	63.5	104.1

**Figure 3:**
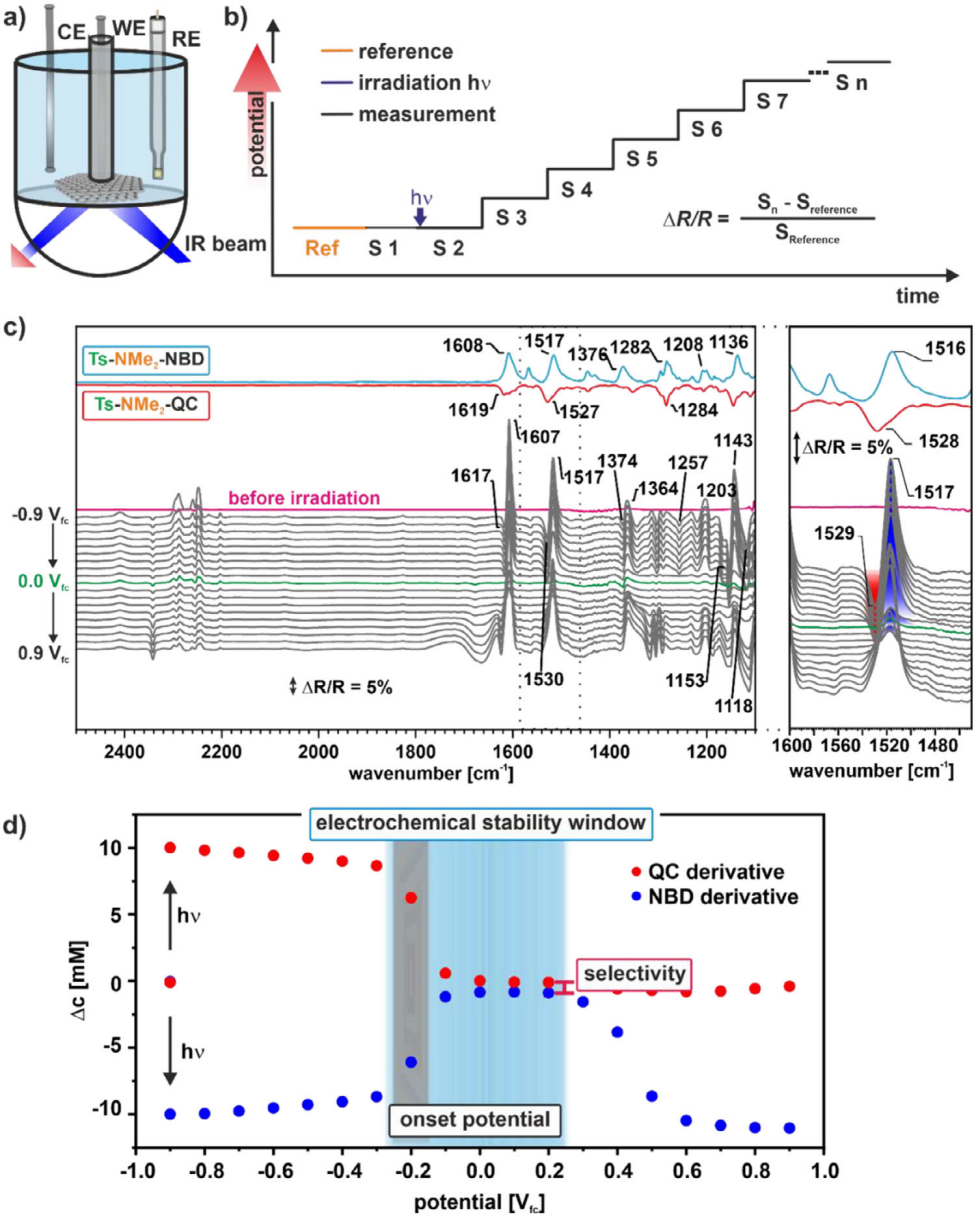
Electrochemically triggered back‐conversion. a) Schematic representation of the PEC‐IRRAS setup; b) experimental procedure; c) IRRAS data of the photochemical conversion and electrochemical back‐conversion of the Ts‐NMe_2_‐NBD/QC system on a HOPG electrode; d) quantitative analysis and important electrocatalytic properties.

To investigate the electrochemically triggered back‐conversion, we first performed photoelectrochemical IR spectroscopy (PEC‐IRRAS) measurements for each of the NBD derivatives. For all measurements, we used a solution of 10 mM NBD derivative in 0.1 M Bu_4_NClO_4_ in MeCN. We used HOPG as the working electrode, graphite as the counter electrode, and Ag/Ag^+^ as the reference electrode (Figure 3a). Note that we refer all measured potentials to the redox potential of ferrocene. We applied a potential of ‐0.9 V_fc_ and recorded a background spectrum. We then irradiated the solution and recorded another spectrum, increased the potential in 100 mV steps, and recorded an IR spectrum at each potential step. For each NBD derivative, the experiment was performed twice to validate its reproducibility. The experimental procedure is shown in Figure 3b.

The corresponding IR spectra of the Ts‐NMe_2_‐NBD/QC system are shown exemplarily in Figure 3c with a zoom into the spectroscopic marker region around 1500 cm^−1^. This band belongs to the ν(CC) + δ(CH) vibration of the phenyl group at the EDG. Note that all compounds investigated have this specific band (see Supporting Information Section 2, Figures S1–S7), which shifts by ∼10 cm^−1^ upon irradiation. We used this band as a spectroscopic marker for all compounds. All spectra shown are difference spectra with respect to the spectrum recorded immediately before irradiation. As a result, negative (oriented downward) and positive (oriented upward) bands indicate formed and consumed species, respectively.

After irradiation, we observe positive bands at 1607, 1517, 1364, 1203, and 1143 cm^−1^. We assign these bands to the ν(CC)_NBD_, ν(CC)_phenyl_, ν(CN), ν(CS), and ν(CH)_methyl_ bands of the Ts‐NMe_2_‐NBD, respectively, based on our peak assignment using DFT. Note that we provide the band assignment of all NBD/QC derivatives studied in this work in Figures S1–S7 and Table S1–S14 in Section 2 of the Supporting Information. We also observe negative bands at 1617, 1530, 1257, 1153, and 1118 cm^−1^, which we attribute to the ν(CC)_QC_, ν(CN), ν(CC)_phenyl_, ν(CO), ν(SO) vibrations of formed Ts‐NMe_2_‐QC. Note that the solvent MeCN has a large CN band at 1450 cm^−1^, which makes this region unusable for evaluation.^[^ ^46^ ^]^ The results indicate the photochemical conversion of Ts‐NMe_2_‐NBD to Ts‐NMe_2_‐QC. When increasing the potential from ‐0.9 to ‐0.3 V_fc_, the bands remain largely unchanged. At ‐0.2 V_fc_ and above, we observe a fast decay of the bands of Ts‐NMe_2_‐QC as well as Ts‐NMe_2_‐NBD. While the bands of Ts‐NMe_2_‐QC disappear completely, a small positive contribution of Ts‐NMe_2_‐NBD remains, as shown exemplarily by the band at 1517 cm^−1^ of the spectroscopic marker. We conclude that Ts‐NMe_2_‐QC is mainly back‐converted to Ts‐NMe_2_‐NBD, while a small fraction of Ts‐NMe_2_‐QC is already decomposed, limiting the selectivity. At 0.3 V_fc_ and above, we observe again an increase of the positive bands assigned to Ts‐NMe_2_‐NBD, indicating the on‐set of the oxidative decomposition of the NBD derivative.

There are certain properties that characterize the electrochemically triggered energy release from QC as this is shown in the quantitative analysis of the spectra (Figure 3d). For the quantitative analysis, we calculated the concentrations of the compounds according to a procedure described in previous work.^[^ ^20^ ^]^ The first property is the onset potential of the oxidation (black window) that initiates the back‐conversion. In the specific example presented here, this is observable at ‐0.2 V_fc_. A detailed description of the procedure used to determine the onset potentials is provided in Section 6 and Figure S15 of the Supporting Information. The second property is the electrochemical stability window of the back‐conversion (indicated as gray area), which is the potential difference between the onset of QC oxidation initiating the back‐conversion and the onset of the NBD oxidation, initiating the decomposition of the MOST. In the specific example of Ts‐NMe_2_‐NBD/QC the potential window is 0.5 V (between ‐0.2 and 0.3 V_fc_). For applications, a large electrochemical stability window is beneficial, as this forgives inaccuracies in applying the potential without decomposing the MOST. The onset potential of the back‐conversion and the stability window can be used to derive the potential range that can be approached in the application to trigger the back‐conversion and has also to match the electrochemical stability window of the solvent used. The last property is the selectivity (S_NBD_ = −Δc_NBD_/Δc_QC_), which is especially important. In Figure 3d, the red bar indicates the loss during back‐conversion, resulting in a limited selectivity of 91.5% for the example shown. Ideally, the QC derivatives are back‐converted without side product formation. This ensures excellent cyclability and a long lifetime of a MOST device without the need for regular replacement of the energy storage material.

**Figure 4:**
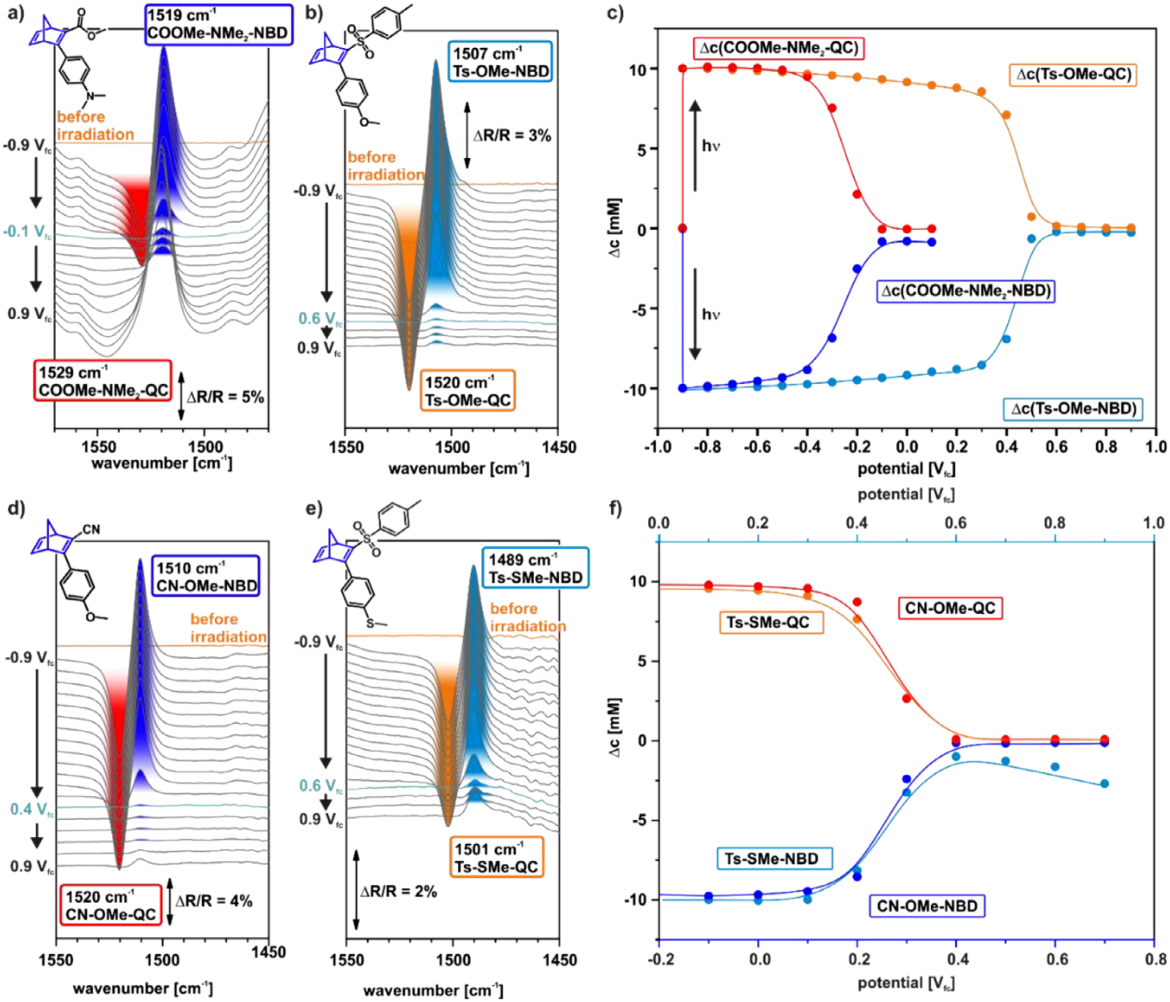
Comparison of the best‐ and worst‐performing molecules in terms of electrochemically triggered back‐conversion, with respect to onset potential and selectivity. a) ν(CC) region as a function of the electrode potential of COOMe‐NMe_2_‐NBD (lowest onset); b) ν(CC) region as a function of the electrode potential of Ts‐OMe‐NBD (highest onset); c) change in concentration of Ts‐OMe‐NBD/QC and COOMe‐NMe_2_‐NBD/QC during the electrochemically triggered back‐conversion; d) ν(CC) region as a function of the electrode potential of CN‐OMe‐NBD (best selectivity); e) ν(CC) region as a function of the electrode potential of Ts‐SMe‐NBD (worst selectivity); f) change in concentration of Ts‐SMe‐NBD/QC and CN‐OMe‐NBD/ QC during the electrochemically triggered back‐conversion.

In Figure 4, we show exemplary spectra of selected NBD derivatives in the frequency range of the spectroscopic marker and the corresponding quantification, which illustrate the effect of different push‐pull functionalities on the onset potential (Figure 4a–c) and the selectivity (Figure 4d–f). The spectra and corresponding quantification of all tested NBD/QC derivatives are provided in the Supporting Information (Section 3, Figures S8–S14). To ensure reproducibility, comparisons with control experiments were included. All measurements showed good agreement with the control data. After irradiation, we observe a shift of the spectroscopic marker to higher wavenumbers for all investigated MOST systems, indicating the successful photoconversion of the NBD isomer (low wavenumber band) to its corresponding QC isomer (high wavenumber band). Quantification shows that within the detection limit of the experiment this conversion is quantitative for all investigated NBD/QC systems. However, drastic differences are observed in the potential dependent spectra. For the COOMe‐NMe_2_‐NBD/QC system (Figure 4a, c), we observe a decrease of the bands assigned to QC (red) and NBD (dark blue) already at ‐0.3 V_fc_, indicating the lowest on‐set potential of the electrochemically induced back‐conversion of all MOST systems studied in this work. In direct contrast is the onset potential for the back‐conversion from QC (orange) to NBD (light blue) of the Ts‐OMe‐NBD/QC system (Figure 4b, c) shifted to 0.4 V_fc_, indicating the NBD/QC derivative with the highest on‐set potential. This is a difference of 0.7 V, which is remarkably high.

Figure 4d–f shows the comparison of the IRRA spectra of the CN‐OMe‐NBD/QC and Ts‐SMe‐NBD/QC, and the corresponding quantitative analysis. For CN‐OMe‐NBD/QC (Figure 4d), above the on‐set potential (0.2 V_fc_), we observe a rapid decay of the bands assigned to QC (1520 cm^−1^) and NBD (1510 cm^−1^), with only traces of the latter band remaining at 0.4 V_fc_, indicating a back‐conversion with high selectivity (98.8%). In direct contrast, after electrochemically induced back‐conversion of Ts‐SMe‐QC (on‐set 0.4 V_fc_, Figure 4e), a significant band of NBD (1489 cm^−1^) remains, indicating decomposition and a significant decrease in selectivity to 90.1%.

**Figure 5:**
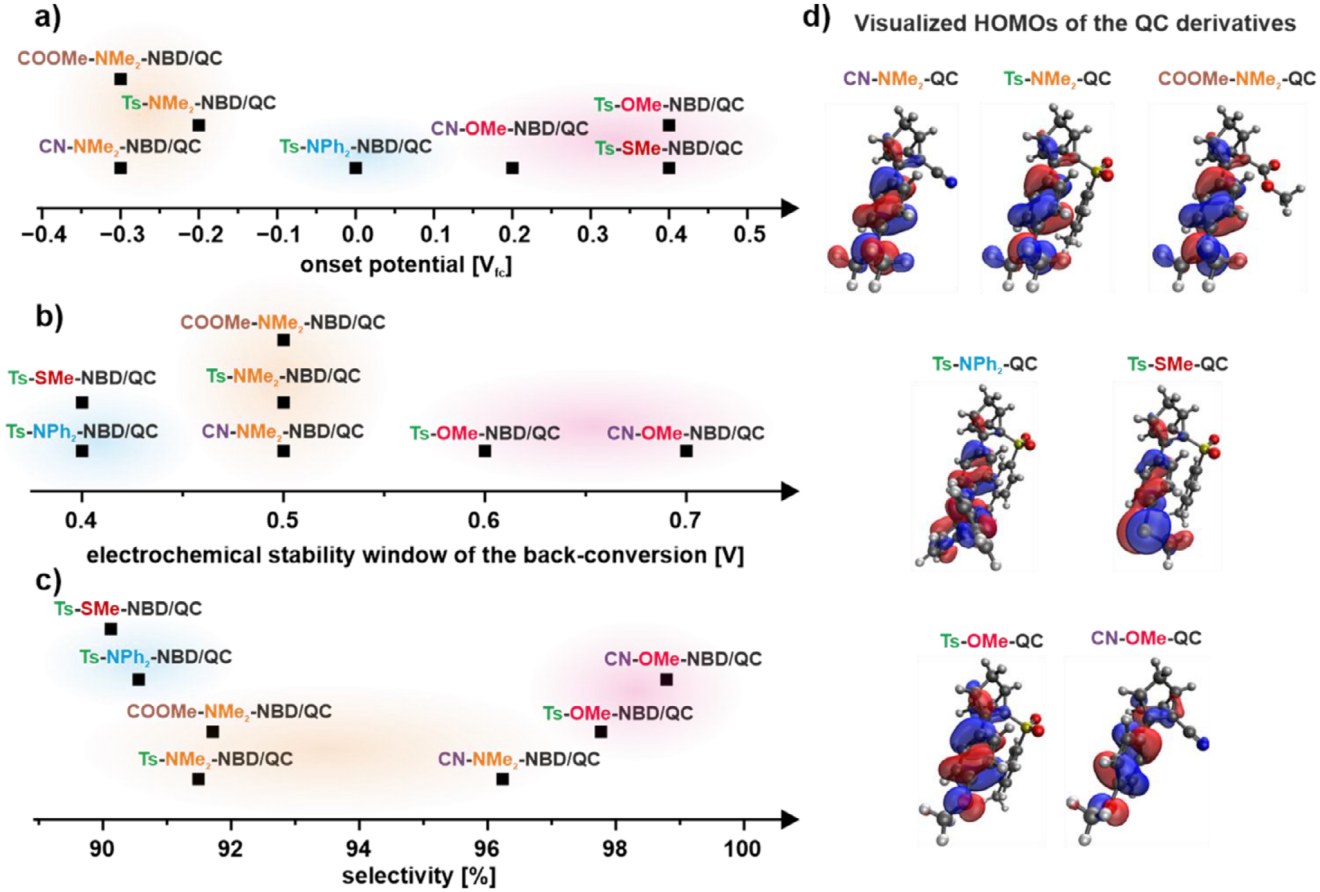
Comparison of the onset potential a), electrochemical stability window b), and selectivity c) of the electrochemically triggered back‐conversion of the investigated compounds; visualized HOMOs (isovalue 0.1) of the investigated QC derivatives d).

In Table 2, we summarize the experimentally determined onset potentials, stability windows, and selectivities for all NBD/QC systems studied in this work. First, we will discuss the trends of the onset potential of the back‐conversion, which are illustrated in Figure 5a. In general, we observe a variation of the onset potential of 0.7 V_fc_. Thus, we observe a distinct influence of the EDG on the onset potential, which follows the order ‐NMe_2_ < ‐NPh_2_ < ‐OMe ≈ ‐SMe, while the effect of the EWG is much smaller.

**Table 2:** Summary of the experimentally determined onset potentials, stability windows, and selectivity.

Compound	CN‐NMe_2_‐NBD/QC	Ts‐NMe_2_‐NBDQC	COOMe‐NMe_2_‐NBD/QC	Ts‐NPh_2_‐NBD/QC	Ts‐SMe‐NBD/QC	Ts‐OMe‐NBD/QC	CN‐OMe‐NBD/QC
Onset back‐conversion [V_fc_]	−0.3	−0.2	−0.3	0.0	0.4	0.4	0.2
Electrochemical stability window of back‐conversion [V]	0.5	0.5	0.5	0.4	0.4	0.6	0.7
Selectivity [%]	96.2	91.5	91.7	90.6	90.1	97.8	98.8

Next, we determined the electrochemical stability window (Figure 5b) and the selectivity (Figure 5c) of the back‐conversion. We observe that the stability window widths vary from 0.4 V (Ts‐NPh_2_‐NBD and Ts‐SMe‐NBD) to 0.7 V (CN‐OMe‐NBD). In the case of the selectivity, we observe the lowest one for the Ts‐SMe‐NBD and the Ts‐NPh_2_‐NBD with 90.1% and 90.6%, respectively. The highest selectivity is observed for the compounds containing an ‐OMe group as EDG (97.8% and 98.8%). Both the stability window and the selectivity depend mainly on the EDG, following the order ‐SMe ≈ ‐NPh_2_ < ‐NMe_2_ < ‐OMe.

To explain the trends of our experimental observations qualitatively, we studied the different compounds by DFT (Table 3, for details see Supporting Information). We emphasize that while these DFT analyses do not aim at a quantitative description, they serve as a useful interpretative tool for understanding the influence of substitution patterns. We also acknowledge that a fully quantitative description would require higher‐level theoretical approaches and multiconfigurational methods.^[^ ^47^, ^48^ ^]^ First, we evaluated the energies of the HOMO and LUMO and from this calculated the corresponding gap. The latter decreases as the strength of the push‐pull system attached to the QC increases. According to our calculations, Ts‐NPh_2_‐QC and Ts‐NMe_2_‐QC have the smallest HOMO‐LUMO gaps and, therefore, are the strongest push‐pull systems. The compounds Ts‐OMe‐QC, CN‐NMe_2_‐QC, COOMe‐NMe_2_‐QC, and Ts‐SMe_2_‐QC show similar HOMO‐LUMO gaps. The compound with the largest HOMO‐LUMO gap is compound CN‐OMe‐QC. We attribute the small HOMO‐LUMO gaps of systems containing amino groups to the strong electron‐donating effect of the amino groups.^[^ ^49^ ^]^ The next step was to calculate the adiabatic ionization potential (IP) of the QC derivatives. We did this by calculating the minimum energies of the QC and QC^+•^ (for exact energies, see Supporting Information) and subtracting them from each other. As expected, these IPs show a similar trend compared to the HOMO energies. The compounds with amino substituents have lower IPs than the other three substituents. We conclude that the strong electron donation of the amine groups raises the HOMO level relatively high compared to the other compounds, thus reducing the energy required for oxidation. The trends observed for the computed IPs (Table 3) fit those observed for the experimental oxidation potential (Table 2 and Figure 5a). Finally, we visualized the HOMOs of the different QC derivatives (Figure 5d). They are all clearly localized on the electron‐rich EDG. We conclude that the EWG is almost irrelevant for the electrochemically induced oxidative back‐conversion, since it is not directly involved in the oxidation. This in turn translates into the strong influence of the EDG alone on the onset potential (Figure 5a).

**Table 3:** Overview of the investigated compounds and their HOMO‐LUMO gaps and IPs.

Compound	CN‐NMe_2_‐NBD/QC	Ts‐NMe_2_‐NBDQC	COOMe‐NMe_2_‐NBD/QC	Ts‐NPh_2_‐NBD/QC	Ts‐SMe‐NBD/QC	Ts‐OMe‐NBD/QC	CN‐OMe‐NBD/QC
HOMO‐LUMO gap of NBDs [eV]	6.20	6.22	6.43	6.17	7.17	6.76	6.69
HOMO‐LUMO gap of QCs [eV]	7.75	7.02	7.81	6.71	7.91	7.62	8.35
IP of the NBD derivative [eV]	6.80	6.57	6.67	6.75	7.18	7.17	7.38
IP of the QC derivative [eV]	6.65	6.73	6.53	6.63	7.33	7.25	7.27

The observed trends in selectivity can be explained by the stabilizing effects of the EDG. It is well established that EDGs stabilize carbocations by donating π‐electrons to the empty p‐orbitals of the cation.^[^ ^49^, ^50^, ^51^, ^52^, ^53^ ^]^ This rationalizes the observed increase in selectivity upon introduction of electron‐donating substituents on the aryl ring of the EDG.^[^ ^42^ ^]^ The different trends observed for the ‐NMe_2_ and ‐OMe moieties can be explained by mesomeric and inductive effects. As previously discussed, ‐NMe_2_ group exhibits a stronger electron delocalization effect (+M effect), thus pushing more electron density into the EDG and, thus, increasing the energy level of the HOMO.^[^ ^54^ ^]^ The ‐OMe moiety in comparison has a greater electronegativity. This leads to a destabilization of the positive charge on the tertiary carbon attached near the oxygen atom and, thus, a higher localization to the QC scaffold. Conversely, the –NMe_2_ substituent more effectively delocalizes the charge toward the heteroatom, rendering the quinone‐like mesomeric resonance structure more significant. This, combined with the stronger overall charge distribution in the aryl moiety, changes the reactivity, leading to more side reactions compared to ‐OMe. Since we could not identify distinct structures of the formed side products, evaluation of a certain ongoing mechanism is not possible at this point.

## 3 Conclusion

In this study, we systematically investigated how common electron‐donating (EDG; attached in the para position to a phenyl group) and electron‐withdrawing (EWG) substituents influence the electrochemically triggered energy release in MOST systems based on the norbornadiene/quadricyclane (NBD/QC) pair. Using a combination of in‐situ photoelectrochemical infrared spectroscopy (PEC‐IRRAS) and computational modeling (DFT), we identified key trends in the onset potential, electrochemical stability, and selectivity of the back‐conversion process.

· *Onset potential*: The onset potential, which determines when the back‐conversion occurs, varies significantly from ‐0.3 to + 0.4 V_fc_. A clear trend emerges based on the strength of the electron‐donating effect of the EDG: −NMe_2_ < −NPh_2_ < −OMe ≈ −SMe. This behavior is explained by the mesomeric (+M) and inductive (+I) effects, where stronger electron donation raises the HOMO energy level and in turn lowers the IP, thereby reducing the energy required for oxidation.
· *Electrochemical stability window*: The electrochemical stability window of the back‐conversion process is defined as the range of potentials between the onset of electrochemically triggered back‐conversion of QC to NBD and the onset of oxidative decomposition of the NBD derivative. For the investigated molecules, the stability window widths vary from 0.4 to 0.7 V and strongly depend on the EDG. The trend follows: −SMe ≈ −NPh_2_ < −NMe_2_ < −OMe.
· *Selectivity*: The selectivity of the electrochemically triggered back‐conversion of the investigated derivatives ranges from 90.1% (Ts‐SMe‐QC) to 98.8% (CN‐OMe‐NBD) and follows a similar trend as the electrochemical stability window. It is attributed to inductive (I effects) and mesomeric effects (M effects). The **− **OMe group, being more electronegative than **− **NMe_2_ or **− **SMe, destabilizes the positive charge near the oxygen atom, leading to better charge localization at the QC. In contrast, the **− **NMe_2_ group facilitates charge delocalization over the phenyl ring, increasing side reactions and reducing selectivity.

Our results show that the choice of EDG in push‐pull NBD/QC systems significantly affects the electrochemically triggered energy release in MOST systems. This systematic substituent study establishes a basis for selecting optimal MOST candidates with tailored electrochemical properties. In addition to providing immediate experimental design guidance, it also opens the door to more in‐depth theoretical work. Building on these results, our ongoing efforts focus on high‐level computational investigations of the switching mechanism and competing side reactions, including methods capable of capturing the multiconfigurational nature of the NBD/QC interconversion. These future studies aim to further deepen our understanding of energy storage and release in electrochemically switchable MOST systems.

## Supporting Information

The authors have cited additional references within the Supporting Information.^[^ ^55^, ^56^, ^57^, ^58^, ^59^, ^60^ ^]^

## Author Contributions

E.F.: Conceptualization, investigation, formal analysis, writing – original draft, N.O.: Investigation, formal analysis, writing – review & editing, D.K.: Resources, writing – review & editing, N.B.: Resources, writing – review & editing, Z.H.: Visualization, writing – review & editing, K.M.‐P., Resources, supervision, funding acquisition, writing – review & editing, H.H. Resources, supervision, funding acquisition, writing – review & editing, A.H.: Resources, supervision, funding acquisition, writing – review & editing, A.D.: Supervision, funding acquisition, writing – review & editing, O.B.: Conceptualization, supervision, funding acquisition, writing – original draft, J.L.: Resources, funding acquisition, writing – review & editing.

## Conflict of Interest

The authors declare no conflict of interest.

## Declaration of Generative AI and AI‐Assisted Technologies in the Writing Process

During the preparation of this work the authors used AI‐assisted technology in order to improve the language. After using this tool, the authors reviewed and edited the content as needed and take full responsibility for the content of the publication.

## Supporting information



Supporting Information

## Data Availability

The data that support the findings of this study are openly available in Zenodo at https://doi.org/10.5281/zenodo.14803619, reference number DOI:10.5281/zenodo.14803619.
