# Supplementary material for: Tuning Electrocatalytic Energy Release in Norbornadiene Based Molecular Solar Thermal Systems Through Substituent Effects
Source: Chemistry. 2025 Aug 6;31(49):e02294. doi: 10.1002/chem.202502294 (PMC12405824; doi:10.1002/chem.202502294)
Supplement: Supplementary file 1 — Supporting Information [file CHEM-31-e02294-s001.pdf]

Supporting Information

Tuning Electrocatalytic Energy Release in Norbornadiene Based Molecular Solar Thermal Systems Through Substituent Effects

Evanie Franz[a] †, Nils Oberhof[b] †, Daniel Krappmann[c], Nicolò Baggi[d, e], Zarah Hussain[a], Kasper Moth-Poulsen[d,e,g,h], Helen Hölzel[e,f], Andreas Hirsch[c], Andreas Dreuw[b], Olaf Brummel[a]*, Jörg Libuda[a]

[a]Interface Research and Catalysis, Erlangen Center for Interface Research and Catalysis, Friedrich-Alexander-Universität Erlangen-Nürnberg, Egerlandstraße 3, 91058 Erlangen, Germany.
[b]Interdisciplinary Center for Scientific Computing, Universität Heidelberg, Im Neuenheimer Feld 205 A, 69120 Heidelberg, Germany.
[c] Chair of Organic Chemistry II, Friedrich-Alexander-Universität Erlangen-Nürnberg, Nikolaus-Fiebiger-Straße 10, 91058 Erlangen, Germany.
[d] The Institute of Materials Science of Barcelona, ICMAB-CSIC, Ballaterra, 08193, Barcelona, Spain.
[e]Department of Chemical Engineering, Universitat Politècnica de Catalunya, EEBE, Eduard Maristany 10-14, 08019 Barcelona, Spain.
[f]Institut Organische Chemie, Justus-Liebig-Universität Gießen, Heinrich-Buff-Ring 17, 35392 Gießen, Germany.
[g] Catalan Institution for Research & Advanced Studies, ICREA, Pg. Lluís Companys 23, 08010 Barcelona, Spain.
[h] Chalmers University of Technology, Department of Chemistry and Chemical Engineering, SE-412 96 Gothenburg, Sweden.

*corresponding author: Dr. Olaf Brummel, olaf.brummel@fau.de

†This author contributed equally and should be also considered as first author

1. Experimental Section

Cleaning: All Teflon and glass ware was stored in sulfuric acid (Merck, Emsure, 98%) with NOCHROMIX® (Sigma Aldrich) overnight. Before use, the equipment was rinsed with ultra-pure water (MilliQ Synergy UV, 18.2 MΩ·cm at 25 °C, TOC < 5 ppb) for 5 times and boiled 3 times in ultra-pure water for 30 min. Afterwards it was dried under rough vacuum overnight.

PEC-IRRAS: To measure PEC-IRRAS, we used a vacuum-based FT-IR spectrometer (Bruker, Vertex 80v) with evacuated optics and a liquid-nitrogen-cooled mercury cadmium telluride (MCT) detector. The photochemical conversion was performed with an UV LED (Seoul Viosys, CUD1AF4D, 310 nm, 30 mW; CUD4AF1B, 340 nm, 55 mW; CUN6AF1B, 365 nm, 820 mW). This was located below an IR and UV transparent CaF_2 (Korth, d = 25 mm) hemisphere. The IR detector was protected from UV light with a KRS-5 filter. All in-situ measurements were measured in reflection mode in thin layer configuration. Potential dependent spectra were recorded with a resolution of 2 cm^{-1}, a scanner velocity of 40 kHz, 128 scans per spectrum (background 256 scans per spectrum) and an acquisition time of 57 s (background 114 s). For all measurements, we used non-polarized light. We recorded transmission spectra using KBr (≥ 99%, FTIR-grade, Sigma Aldrich) pellets. For the transmission spectra of the QC isomers, we irradiated the KBr pellet with the corresponding LED until the resulting IR spectra did not change.

PEC-IRRAS experiments were performed using a HOPG crystal (MikroMasch, ZYA, 0.4° mosaic spread) as WE, which was cleaned prior to each measurement by cleavage using scotch tape. We used a solution of 10 mM NBD derivative in 0.1 M Bu_4NClO_4 (Sigma Aldrich, ≥99.0%) as supporting electrolyte in MeCN (Sigma Aldrich, 99.999% trace metals basis). The potential was applied using a commercial potentiostat (Gamry, Reference 600) with a three-electrode setup. We used a graphite rod as counter electrode (CE) and an Ag/Ag^+ (0.01 M $AgNO_3$ with 0.1 M Bu_4NClO_4 in MeCN) electrode as reference electrode (RE). The RE was calibrated before and after each measurement day versus the redox potential of ferrocene (Alfa Aesar, 99.5%), determined by cyclic voltammetry. Note that, in this work, we referred all potentials to the redox potential of the ferrocene pair (V_{fc}).

DFT calculations:

All calculations were performed using the Qchem 5.3 software package.[1] Ground state optimizations were done at the density functional theory (DFT) level, utilizing CAM-B3LYP (a range separated hybrid exchange correlation (xc)-functional) with the 6-311G* basis set and Grimme's D3(BJ) dispersion correction.[2–4] Frequency calculations were employed to verify the geometries as local minima with zero imaginary frequencies. The $QC^{+\bullet}$ species were optimized in an unrestricted DFT framework with the same functional, basis set and dispersion correction combination. For the quantitative description of the isomerization processes of NBD/QC multi-reference methodology has previously been employed.[5,6] Here, we chose to circumvent the extensive computational demand of such methods by employing DFT calculations for the ground-state properties of the investigated systems, since we do investigate qualitative trends.

Synthesis of NBDs:

CN-NMe$_2$-NBD:

The synthesis was modified from a previously published method. For further details, we refer to literature.[7]

A pressure tube was charged with the alkyne precursor (425.6 mg, 2.50 mmol), toluene (1.25 mL), and freshly distilled cyclopentadiene (230 mg, 3.48 mmol). The tube was sealed and heated to 150°C for 48 h. The solvent was evaporated, and the crude product was purified by column chromatography (dichloromethane/petroleum ether, 1/1 v:v). After evaporation of the solvent, the solid was washed with diethyl ether and hexane to yield the pure NBD (273.3 mg, 1.16 mmol, 46%) as a yellow solid.

CN-OMe-NBD:

The synthesis was carried out based on a previously published method.[8]

Ts-NMe$_2$-NBD:

2-bromo-3-tosylbicyclo[2.2.1]hepta-2,5-diene (1.54 mmol, 500 mg, 1.0 equiv.), *N*,*N*-dimethyl-4-(4,4,5,5-tetramethyl-1,3,2-dioxaborolan-2-yl)aniline (1.85 mmol, 457 mg, 1.2 equiv.), K_2CO_3 (4.6. equiv.), RuPhos (0.1 equiv.), and $Pd(OAc)_2$ (5 mol%) were placed in a flame dried pressure

tube under a nitrogen atmosphere. A previously prepared mixture of toluene (10 mL) and H_2O (2.5 mL) was purged with N_2 for 30 min before being added to the vial. The obtained biphasic reaction mixture was heated at 80°C for 22 h. Purification was achieved *via* automized flash column chromatography using a mixture of 20 % EtOAc in hexanes as eluent yielding a yellow solid (136 mg, 0.371 mmol, 24%).

For further details, we refer to literature.[9]

COOMe-NMe2-NBD:

The synthesis was adapted from literature. Methyl 3-(4-(dimethylamino)phenyl)propiolate (400 mg, 1.97 mmol, 1.0 equiv.) and freshly cracked cyclopentadiene (211 µL, 2.56 mmol, 1.3 equiv.) were put in a 10 ml microwave vial and dissolved in toluene (3 mL). The mixture was purged with nitrogen for 10 minutes and subsequently heated to 190°C for 5 h using microwave irradiation. The reaction was monitored with GC/MS every 1 h and stopped after 60% conversion was achieved. Longer reaction time and higher temperature led to additional DIELS-ALDER adducts. After complete reaction time, the mixture was cooled to room temperature and the solvent removed. Purification was achieved *via* automized flash column chromatography (hexanes/ethyl acetate, 4/1 v:v) yielding the title compound as a yellow solid (151 mg, 0.561 mmol, 28 %).
For further details, we refer to literature.[10]

Ts-NPh2-NBD:

2-bromo-3-tosylbicyclo[2.2.1]hepta-2,5-diene (1.54 mmol, 500 mg, 1.0 equiv.), (4-(diphenylamino)phenyl)boronic acid (1.85 mmol, 534 mg, 1.2 equiv.), K_2CO_3 (4.6. equiv.), RuPhos (0.1 equiv.), and $Pd(OAc)_2$ (5 mol%) were placed in a flame dried pressure tube under a nitrogen atmosphere. A previously prepared mixture of toluene (10 mL) and H_2O (2.5 mL) was purged with N_2 for 30 min before being added to the vial. The obtained biphasic reaction mixture was heated at 80°C for 20 h. Purification was achieved *via* automized flash column chromatography using a mixture of 20 % EtOAc in hexanes as eluent yielding a yellow solid (469 mg, 0.958 mmol, 62 %).

For further details, we refer to literature.[9]

Ts-SMe-NBD:

2-bromo-3-tosylbicyclo[2.2.1]hepta-2,5-diene (1.23 mmol, 400 mg, 1.0 equiv.), 4-(methylthio) phenyl)boronic acid (1.48 mmol, 248 mg, 1.2 equiv.), K_2CO_3 (4.6. equiv.), RuPhos (0.1 equiv.), and $Pd(OAc)_2$ (5 mol%) were placed in a flame dried pressure tube under a nitrogen atmosphere. A previously prepared mixture of toluene (10 mL) and H_2O (2.5 mL) was purged with N_2 for 30 min before being added to the vial. The obtained biphasic reaction mixture was heated at 80°C for 18 h. Purification was achieved *via* automized flash column chromatography using a gradient of 20 → 40 % EtOAc in hexanes yielding an orange solid (195 mg, 0.525 mmol, 43 %).

For further details, we refer to literature.[9]

Ts-OMe-NBD:

2-bromo-3-tosylbicyclo[2.2.1]hepta-2,5-diene (1.54 mmol, 500 mg, 1.0 equiv.), 4-(methoxy-phenyl)boronic acid (1.85 mmol, 281 mg, 1.2 equiv.), K_2CO_3 (4.6. equiv.), RuPhos (0.1 equiv.), and $Pd(OAc)_2$ (5 mol%) were submitted in a flame dried, nitrogen containing pressure tube. A previously prepared mixture of toluene (10 mL) and H_2O (2.5 mL) was purged with N_2 for 30 min before being added to the vial. The obtained biphasic reaction mixture was heated at 80 °C for 6 h. Purification was achieved *via* automized flash column chromatography using a mixture of 20 % EtOAc in hexanes as eluent yielding an orange oil (491 mg, 1.39 mmol, 90 %).

For further details, we refer to literature.[9]

2. Assignment of the IR bands to its vibrational modes

We analyzed the IR spectra of the NBD and QC derivatives by comparing transmission IR spectra and simulated IR spectra from DFT. For the NBD transmission spectra, the NBD derivative was embedded in a KBr matrix. For the QC derivative, the KBr palette was irradiated until the spectrum no longer changed. Both the experimental and the simulated spectra are shown below. In the analysis in the main text, we focus on two characteristic bands that allow us to identify the two photoisomers by in-situ IR spectroscopy. These are the ν(CC)$_{phenyl}$ and δ(CH)$_{phenyl}$ bands around 1500 cm^{-1} (see visualization of vibrational modes). The bands show characteristic shifts of about 10 cm^{-1} upon conversion from NBD to QC. As a result, this band is well suited as a spectroscopic marker and allows us to obtain quantitative information about the concentration of the species.

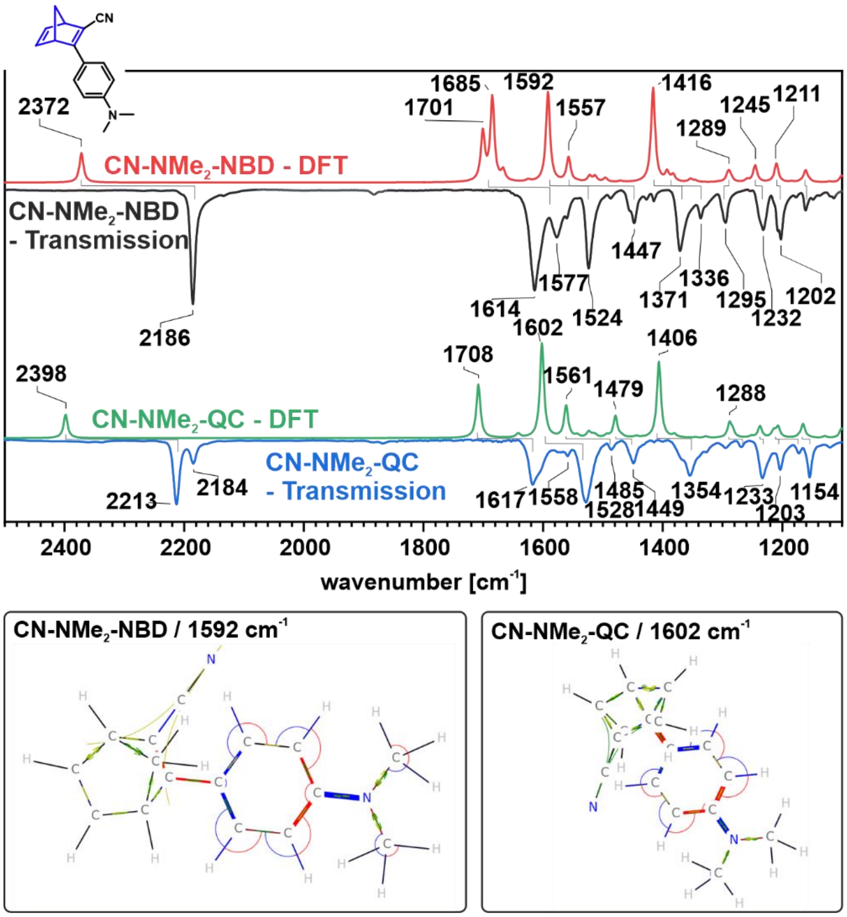

Figure S1: IR spectra of CN-NMe$_2$-NBD. Transmission IR spectra of CN-NMe$_2$-NBD and CN-NMe$_2$-QC and the corresponding spectra calculated by DFT; the spectroscopic marker vibrations ν(CC)$_{phenyl}$ and δ(CH)$_{phenyl}$ are visualized by QVibeplot.[11]

Table S1: Band assignment for CN-NMe$_2$-NBD based on transmission spectra and DFT calculations and visualization of the vibrational modes using QVibeplot.[11]

ν_{exp} [cm^{-1}]	ν_{DFT} [cm^{-1}]	vibrational modes	2D representations of the vibrational modes
2186	2372	ν(CN)	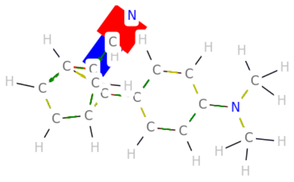
1577	1701	$\nu(CC)_{phenyl}$, $\delta(CH)_{phenyl}$, $\nu(CC)_{NBD}$	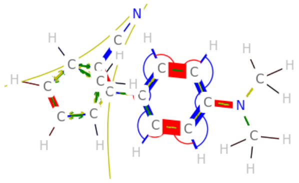
1524	1592	$\nu(CC)_{phenyl}$, $\delta(CH)_{phenyl}$, ν(CN)	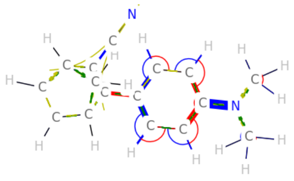
1447	1557	$\delta(CH)_{methyl}$	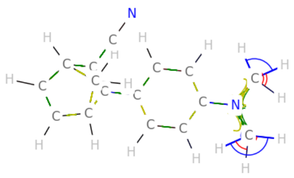

1371	1416	$\nu(CN)$, $\nu(CC)_{phenyl,}$ $\delta(CH)_{methyl}$	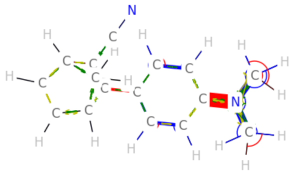
1336	1393	$\nu(CC)_{NBD,}$ $\delta(CH)_{phenyl}$	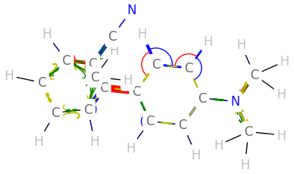
1295	1289	$\nu(CC)_{phenyl,}$ $\nu(CN)_{,}$ $\delta(CH)_{NBD}$	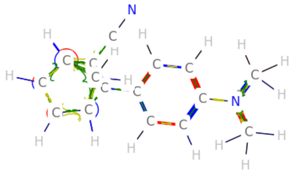
1232	1245	$\nu(CC)_{phenyl,}$ $\delta(CH)_{phenyl}$	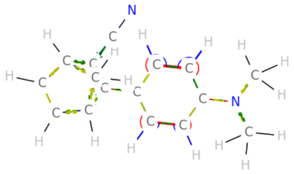
1202	1211	$\nu(CN)$, $\nu(CC)_{phenyl,}$ $\delta(CH)_{methyl}$	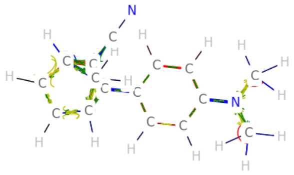

1163	1161	ν(CN), δ(CH)$_{methyl}$	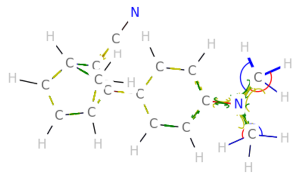

Table S2: Band assignment for CN-NMe$_2$-QC based on transmission spectra and DFT calculations and visualization of the vibrational modes using QVibeplot.[11]

ν_{exp} [cm^{-1}]	ν_{DFT} [cm^{-1}]	vibrational modes	2D representations of the vibrational modes
2213	2398	ν(CN)	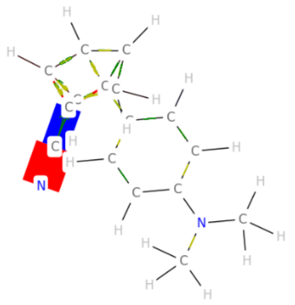
1617	1708	ν(CN), ν(CC)$_{phenyl,}$ δ(CH)$_{phenyl}$	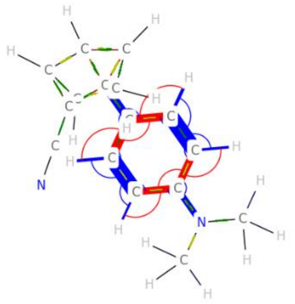
1528	1602	ν(CN), ν(CC)$_{phenyl,}$ δ(CH)$_{phenyl}$	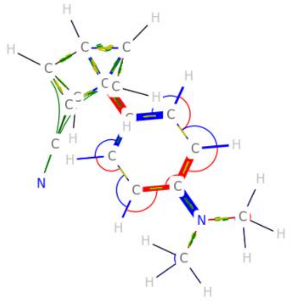
1485	1561	δ(CH)$_{methyl}$	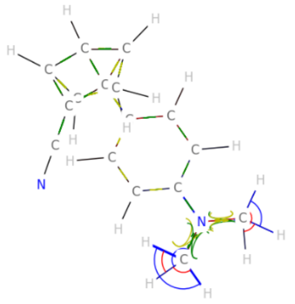

1449	1479	$\nu(CC)_{QC,}$ $\nu(CN)$, $\delta(CH)_{phenyl,}$ $\delta(CH)_{methyl}$	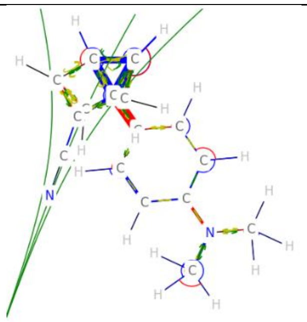
1354	1406	$\nu(CN)$, $\delta(CH)_{phenyl,}$ $\delta(CH)_{methyl}$	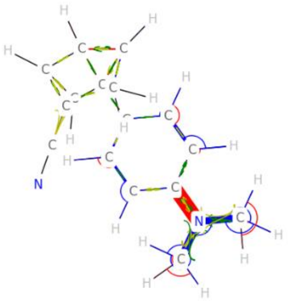
1288	1233	$\nu(CH)_{phenyl}$, $\delta(CH)_{phenyl}$	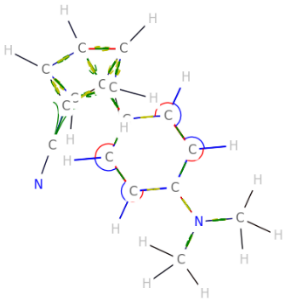
1234	1203	$\nu(CC)_{QC}$, $\nu(CH)_{QC,}$ $\delta(CH)_{QC}$	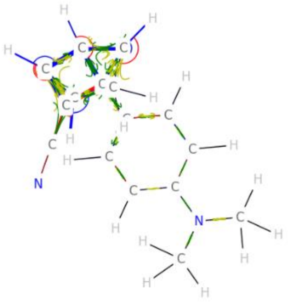
1205	1154	$\nu(CH)_{methyl,}$ $\delta(CH)_{methyl}$	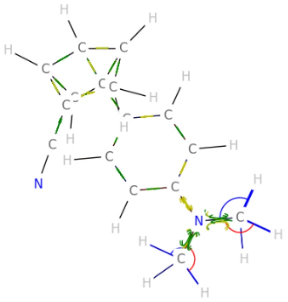

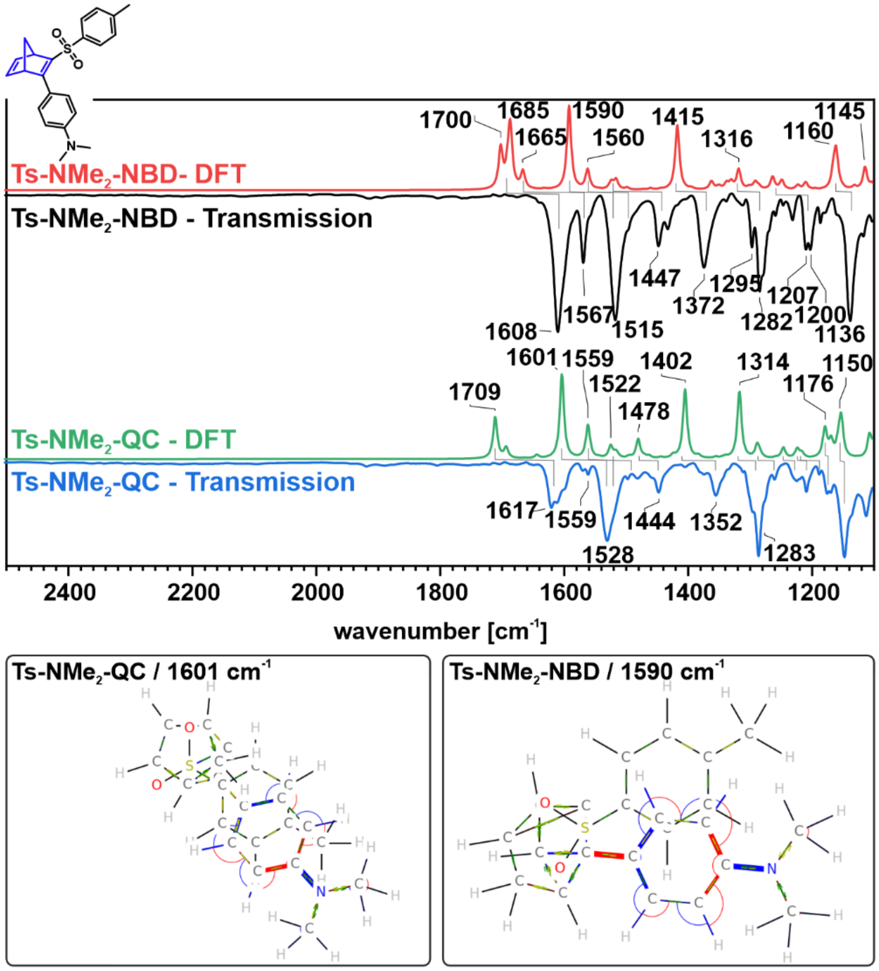

Figure S2: IR spectra of Ts-NMe$_2$-NBD. Transmission IR spectra of Ts-NMe$_2$-NBD and Ts-NMe$_2$-QC and the corresponding spectra calculated by DFT; the spectroscopic marker vibrations $\nu(CC)_{phenyl}$ and $\delta(CH)_{phenyl}$ are visualized by QVibeplot.[11]

Table S3: Band assignment for Ts-NMe_2-NBD based on transmission spectra and DFT calculations and visualization of the vibrational modes using QVibeplot.[11]

ν_{exp} [cm^{-1}]	ν_{DFT} [cm^{-1}]	vibrational modes	2D representations of the vibrational modes
1608	1700	$\nu(CC)_{phenyl,}$ $\delta(CH)_{phenyl,}$ $\nu(CC)_{NBD}$	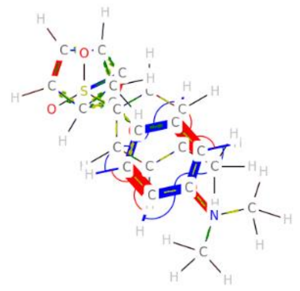
1567	1665	$\nu(CC)_{NBD,}$ $\delta(CH)_{NBD}$	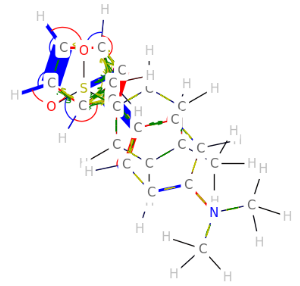
1515	1590	$\nu(CC)_{phenyl,}$ $\delta(CH)_{phenyl}$	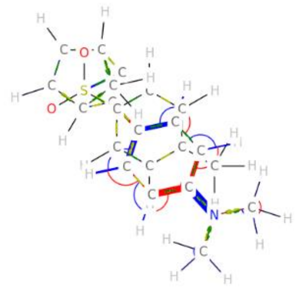
1495	1515	$\nu(CN)_{,}$ $\delta(CH)_{methyl}$	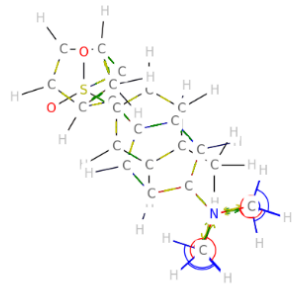

1447	1496	ν(CC)$_{phenyl}$, δ(CH)$_{phenyl}$, δ(CH)$_{methyl}$	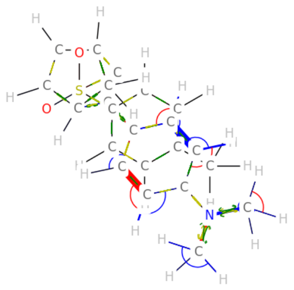
1372	1415	ν(CN), δ(CC)$_{phenyl}$, δ(CH)$_{methyl}$	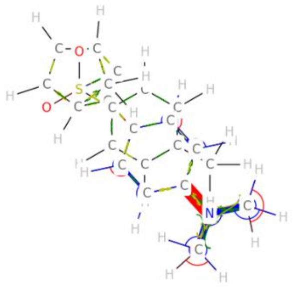
1282	1316	ν(SO), δ(CC)$_{phenyl}$, δ(CH)$_{tosyl}$	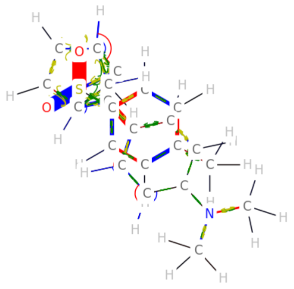
1207	1247	ν(CS), ν(CO), δ(CH)$_{tosyl}$, ν(CH)$_{tosyl}$	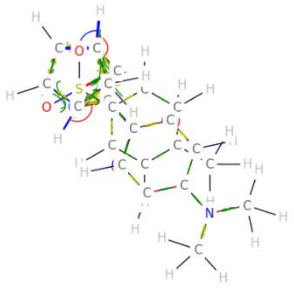
1136	1160	ν(CH)$_{methyl}$, δ(CH)$_{methyl}$	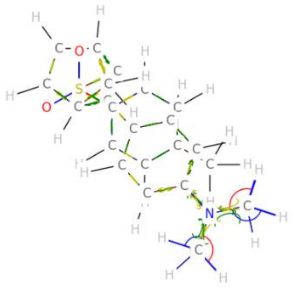

Table S4: Band assignment for Ts-NMe_2-QC based on transmission spectra and DFT calculations and visualization of the vibrational modes using QVibeplot.[11]

ν_{exp} [cm^{-1}]	ν_{DFT} [cm^{-1}]	vibrational modes	2D representations of the vibrational modes
1617	1709	$\nu(CC)_{QC}$, $\delta(CH)_{phenyl}$	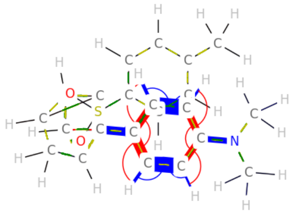
1559	1601	$\nu(CC)_{QC}$, $\nu(CN)$, $\delta(CH)_{phenyl}$	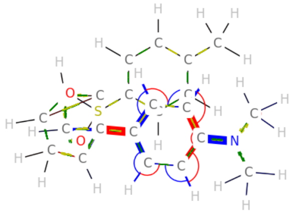
1528	1559	$\nu(CN)$, $\delta(CH)_{methyl}$	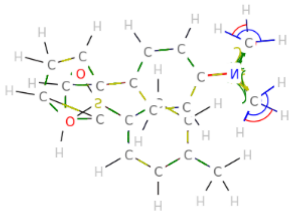
1493	1522	$\nu(CH)_{methyl}$, $\delta(CH)_{methyl}$	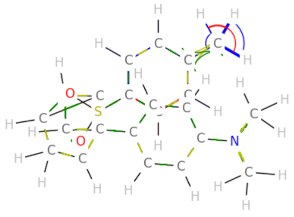

1444	1478	ν(CC), ν(CN), δ(CH)	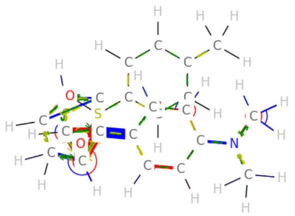
1352	1402	ν(CN), $\delta(CH)_{methyl}$	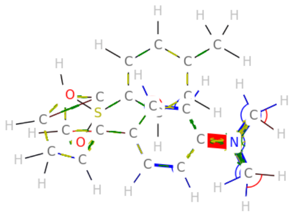
1283	1314	ν(CO), ν(SO). $\delta(CH)_{tosyl}$	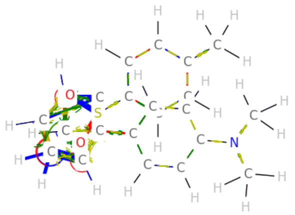
1255	1286	$\nu(CC)_{phenyl}$, ν(CO), ν(CN), $\delta(CH)_{tosyl}$	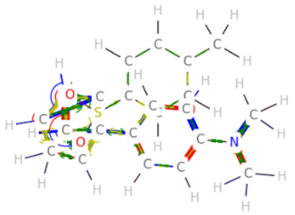
1226	1244	$\delta(CH)_{phenyl}$	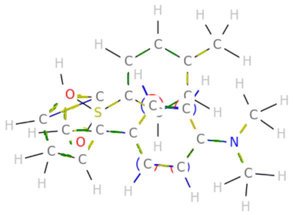

1212	1221	δ(CH)$_{tosyl}$	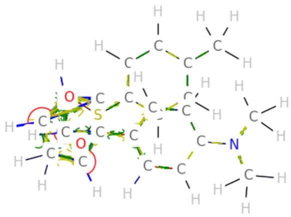
1165	1176	ν(CO), ν(CC)$_{phenyl}$	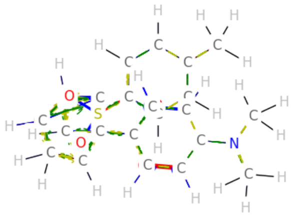
1134	1150	ν(SO), ν(SC), ν(CC)$_{phenyl,}$ δ(CH)	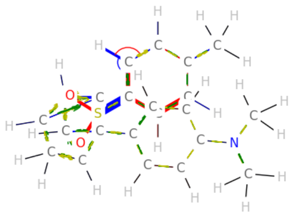

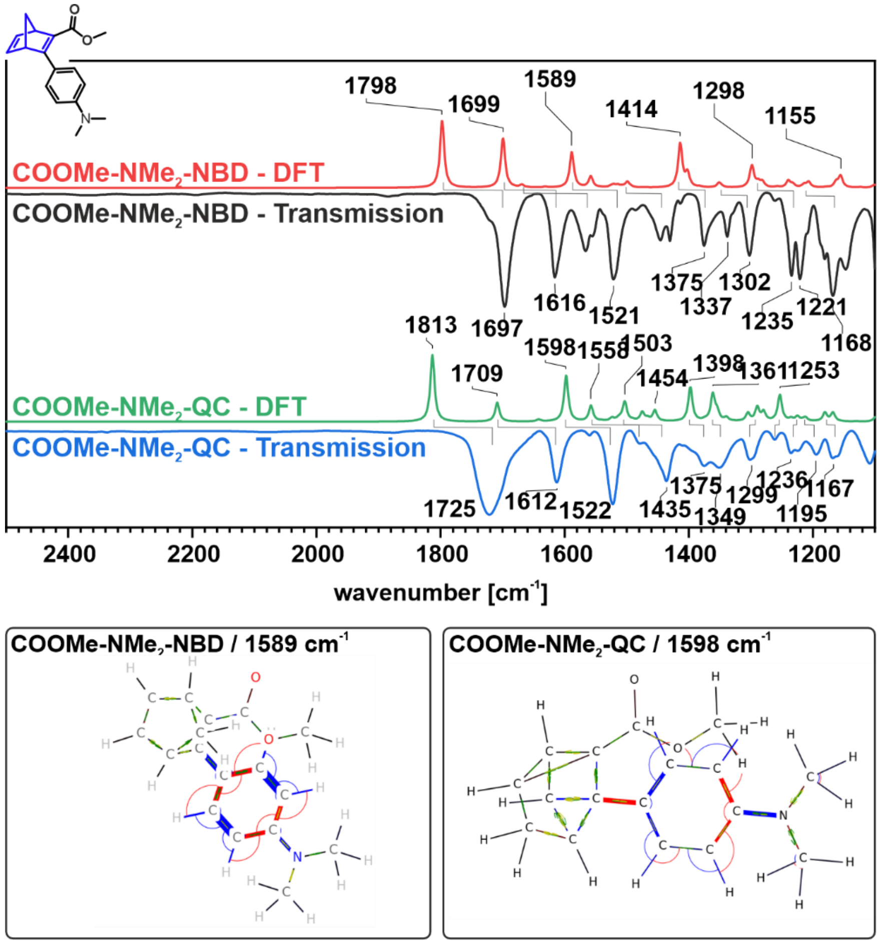

Figure S3: IR spectra of COOMe-NMe$_2$-NBD. Transmission IR spectra of COOMe-NMe$_2$-NBD and COOMe-NMe$_2$-QC and the corresponding spectra calculated by DFT; the spectroscopic marker vibrations $\nu(CC)_{phenyl}$ and $\delta(CH)_{phenyl}$ are visualized by QVibeplot.[11]

Table S5: Band assignment for COOMe-NMe_2-NBD based on transmission spectra and DFT calculations and visualization of the vibrational modes using QVibeplot.[11]

ν_{exp} [cm^{-1}]	ν_{DFT} [cm^{-1}]	vibrational modes	2D representations of the vibrational modes
1697	1798	ν(CO)	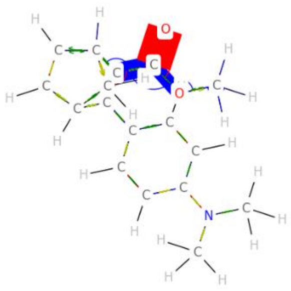
1616	1699	$\nu(CC)_{phenyl}$, $\delta(CH)_{phenyl}$	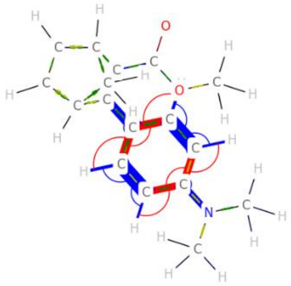
1562	1664	$\nu(CC)_{NBD,}$ $\delta(CH)_{NBD}$	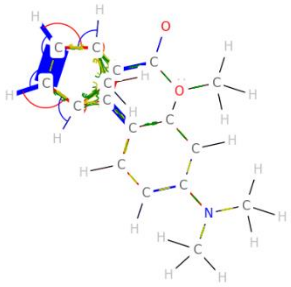
1521	1589	$\nu(CC)_{phenyl}$, ν(CN), $\delta(CH)_{phenyl}$	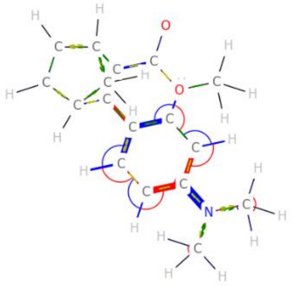

1442	1500	ν(CO), δ(CH)$_{methyl}$	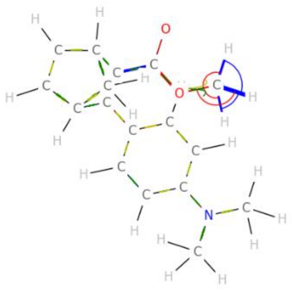
1375	1414	ν(CC)$_{phenyl}$, ν(CN), ν(CO), δ(CH)$_{methyl}$	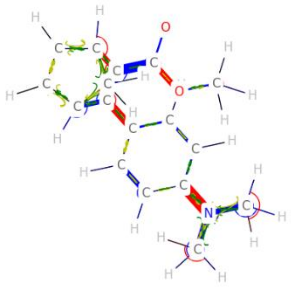
1302	1343	ν(CC)$_{phenyl}$, ν(CN), δ(CH)$_{phenyl}$	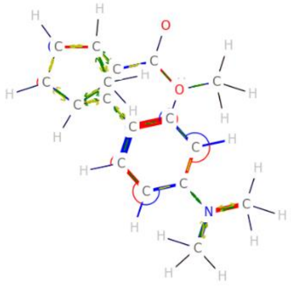
1235	1298	ν(CC)$_{NBD}$, ν(CO), δ(CH)$_{NBD}$	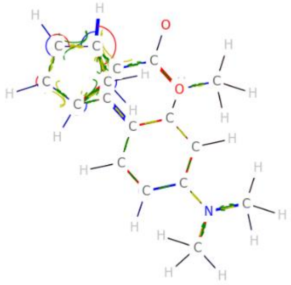
1168	1205	ν(CC)$_{NBD}$, ν(CH)$_{NBD}$, δ(CH)$_{NBD}$	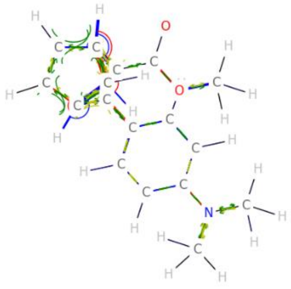

Table S6: Band assignment for COOMe-NMe$_2$-QC based on transmission spectra and DFT calculations and visualization of the vibrational modes using QVibeplot.[11]

ν_{exp} [cm^{-1}]	ν_{DFT} [cm^{-1}]	vibrational modes	2D representations of the vibrational modes
1725	1813	ν(CO)	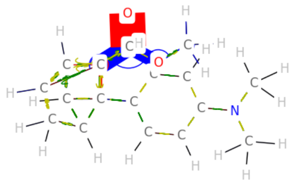
1612	1709	$\nu(CC)_{phenyl}$, ν(CO), $\delta(CH)_{phenyl}$	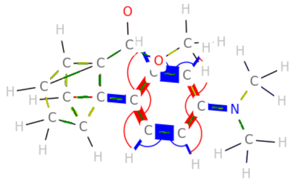
1522	1598	$\nu(CC)_{phenyl}$, ν(CN), $\delta(CH)_{phenyl}$	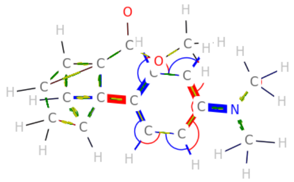
1435	1503	$\nu(CC)_{QC}$, ν(CO), $\delta(CH)_{methyl}$	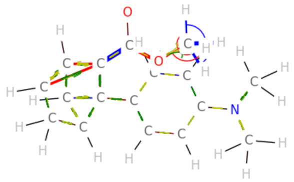

1375	1398	$\nu(CN)$, $\delta(CH)_{methyl}$	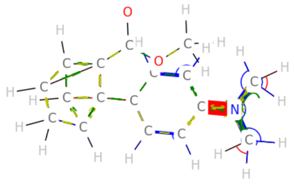
1349	1361	$\nu(CC)_{QC}$, $\nu(CO)$, $\nu(CH)$, $\delta(CH)_{QC}$	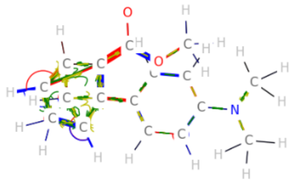
1299	1287	$\nu(CC)_{QC}$, $\nu(CO)$, $\nu(CC)_{phenyl,}$ $\nu(CH)_{QC}$, $\delta(CH)_{QC}$	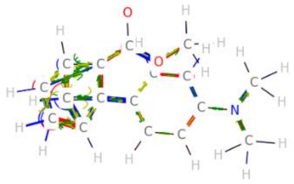
1236	1213	$\nu(CN)$, $\delta(CH)_{methyl}$	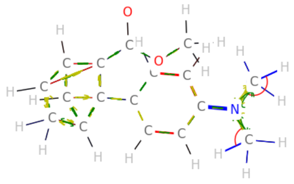
1195	1197	$\nu(CH)_{methyl}$, $\delta(CH)_{methyl}$	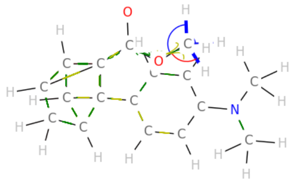

1167	1172	δ$(CH)_{methyl}$	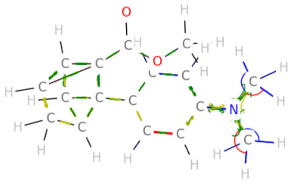

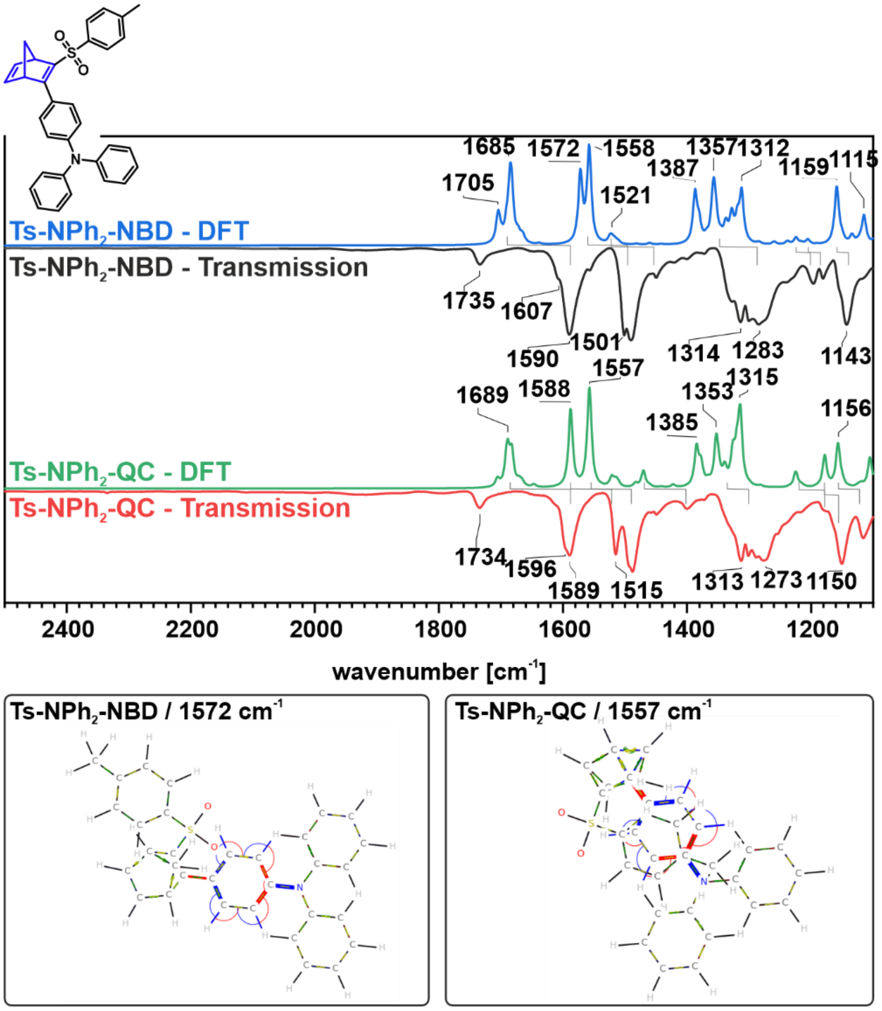

Figure S4: IR spectra of Ts-NPh2-NBD. Transmission IR spectra of Ts-NPh2-NBD and Ts-NPh2-QC and the corresponding spectra calculated by DFT; the spectroscopic marker vibrations ν$(CC)_{phenyl}$ and δ$(CH)_{phenyl}$ are visualized by QVibeplot.[11]

Table S7: Band assignment for Ts-NPh$_2$-NBD based on transmission spectra and DFT calculations and visualization of the vibrational modes using QVibeplot.[11]

ν_{exp} [cm^{-1}]	ν_{DFT} [cm^{-1}]	vibrational modes	2D representations of the vibrational modes
1607	1705	$\nu(CC)_{phenyl}$, $\delta(CH)_{phenyl}$	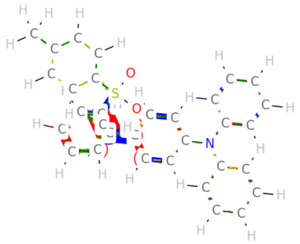
1501	1558	$\nu(CC)_{phenyl}$, $\delta(CH)_{phenyl}$	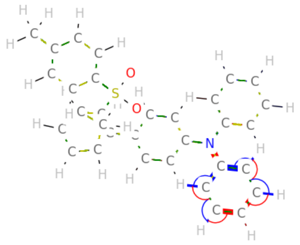
1450	1521	$\nu(CH)$	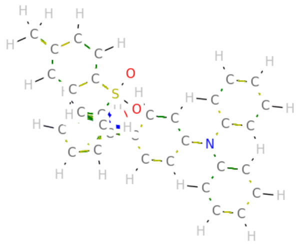
1283	1357	$\nu(CC)_{phenyl}$, $\nu(CN)$, $\delta(CH)_{phenyl}$	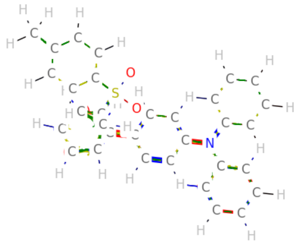

1221	1193	$\delta(CH)_{phenyl}$	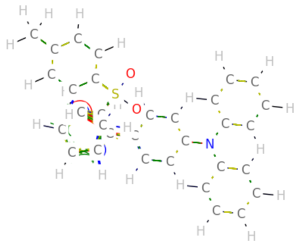
1202	1183	$\nu(CC)_{phenyl}$	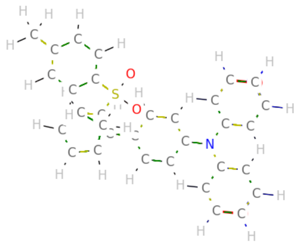
1143	1159	$\nu(CS)$, $\nu(SO)$, $\nu(CC)$	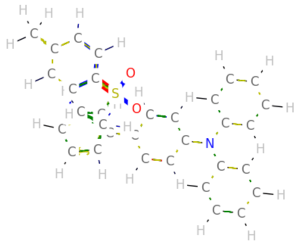

Table S8: Band assignment for Ts-NPh_2-QC based on transmission spectra and DFT calculations and visualization of the vibrational modes using QVibeplot.[11]

ν_{exp} [cm^{-1}]	ν_{DFT} [cm^{-1}]	vibrational modes	2D representations of the vibrational modes
1596	1689	$\nu(CC)_{phenyl}$, $\delta(CH)_{phenyl}$	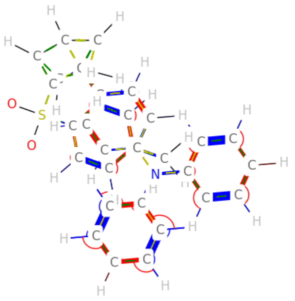
1515	1588	$\nu(CC)_{phenyl}$, $\nu(CN)$, $\delta(CH)_{phenyl}$	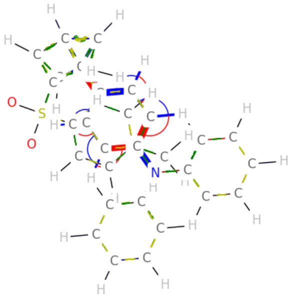
1488	1557	$\nu(CC)_{phenyl}$, $\delta(CH)_{phenyl}$	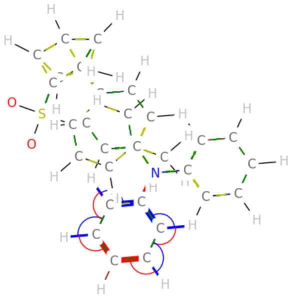
1309	1468	$\nu(CC)_{QC}$, $\delta(CH)_{QC}$, $\delta(CH)_{phenyl}$	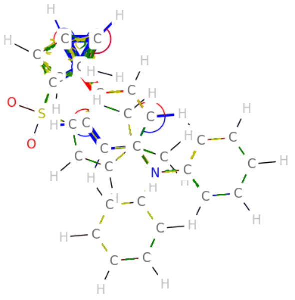

1273	1353	$\nu(CC)_{phenyl}$, $\nu(CN)$, $\delta(CH)_{phenyl}$	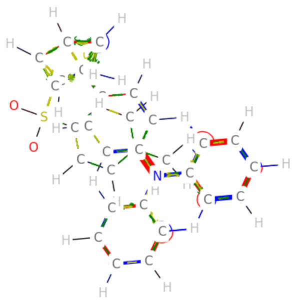
1176	1223	$\nu(CC)_{QC}$	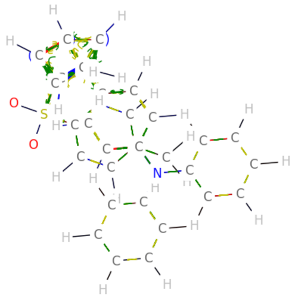
1150	1174	$\nu(CC)_{QC}$, $\nu(SO)$, $\nu(CS)$, $\delta(CH)_{phenyl}$	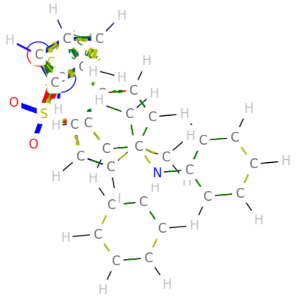
1117	1156	$\nu(SO)$, $\nu(CS)$, $\nu(CC)$	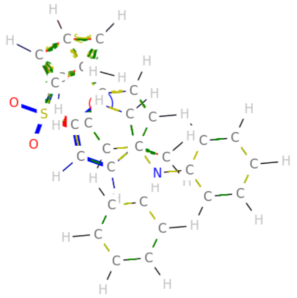

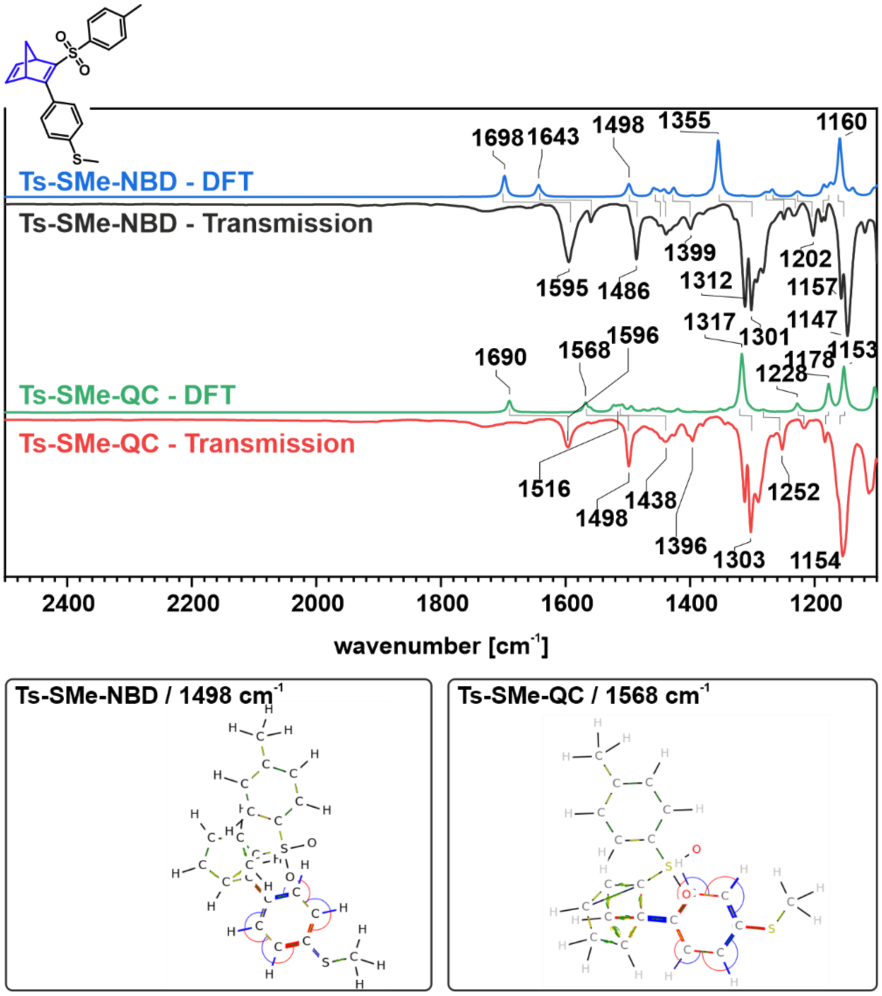

Figure S5: IR spectra of Ts-SMe-NBD. Transmission IR spectra of Ts-SMe-NBD and Ts-SMe-QC and the corresponding spectra calculated by DFT; the spectroscopic marker vibrations $\nu(CC)_{phenyl}$ and $\delta(CH)_{phenyl}$ are visualized by QVibeplot.[11]

Table S9: Band assignment for Ts-SMe-NBD based on transmission spectra and DFT calculations and visualization of the vibrational modes using QVibeplot.[11]

ν_{exp} [cm^{-1}]	ν_{DFT} [cm^{-1}]	vibrational modes	2D representations of the vibrational modes
1595	1698	$\nu(CC)_{phenyl}$, $\nu(CS)$, $\delta(CH)_{phenyl}$	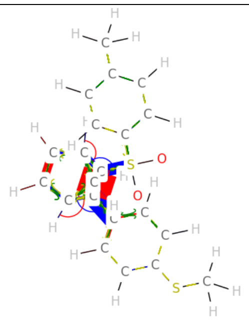
1561	1643	$\nu(CC)_{phenyl}$, $\delta(CH)_{phenyl}$	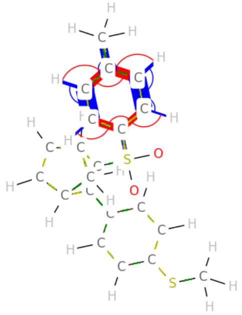
1486	1498	$\nu(CC)_{phenyl}$, $\delta(CH)_{phenyl}$	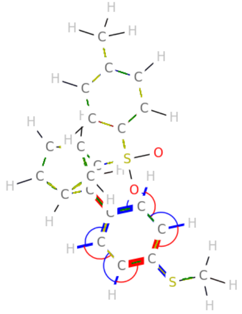
1451	1461	$\delta(CH)_{methyl}$	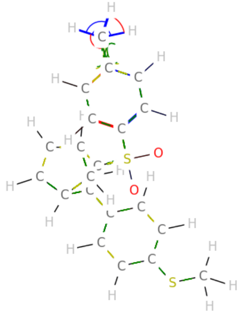

1141	1448	$\nu(CH)_{phenyl}$, $\delta(CH)_{phenyl}$	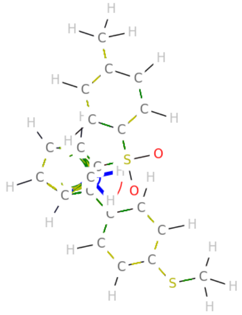
1426	1399	$\nu(CC)_{phenyl}$, $\delta(CH)_{methyl}$	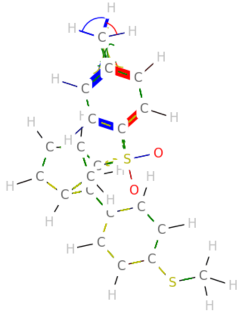
1301	1355	$\nu(SO)$	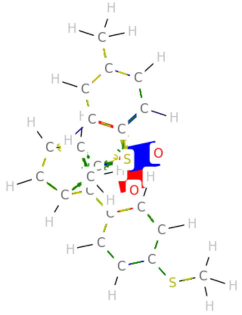
1248	1282	$\nu(CC)_{phenyl}$, $\delta(CH)_{phenyl}$	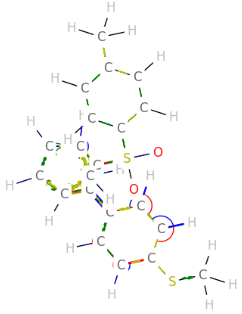
1236	1271	$\nu(CC)_{phenyl}$, $\delta(CH)_{phenyl}$	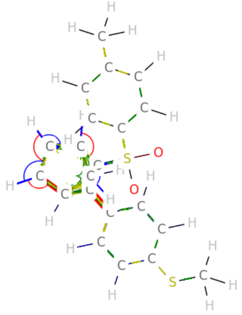

1202	1229	ν(CC)$_{phenyl}$, δ(CH)$_{phenyl}$	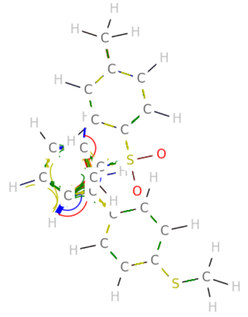
1182	1182	δ(CH)$_{phenyl}$	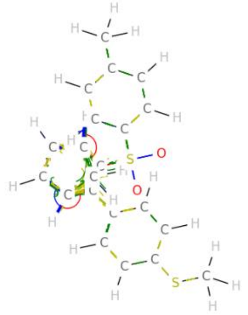
1153	1160	ν(SO), ν(CS)	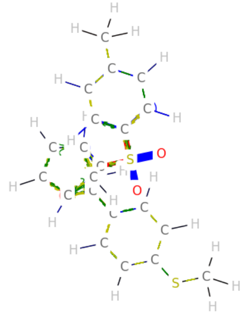

Table S10: Band assignment for Ts-SMe-QC based on transmission spectra and DFT calculations and visualization of the vibrational modes using QVibeplot.[11]

ν_{exp} [cm^{-1}]	ν_{DFT} [cm^{-1}]	vibrational modes	2D representations of the vibrational modes
1516	1690	$\nu(CC)_{phenyl}$, $\delta(CH)_{phenyl}$	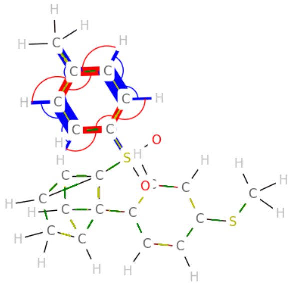
1498	1568	$\nu(CC)_{phenyl}$, $\nu(CO)$, $\delta(CH)_{phenyl}$	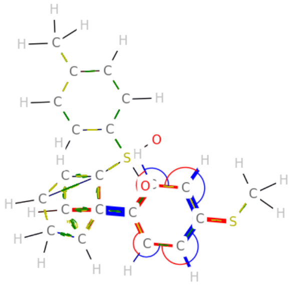
1438	1496	$\delta(CH)_{methyl}$	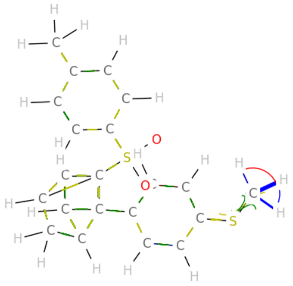
1303	1317	$\nu(CC)_{phenyl}$, $\nu(SO)$, $\delta(CH)_{phenyl}$	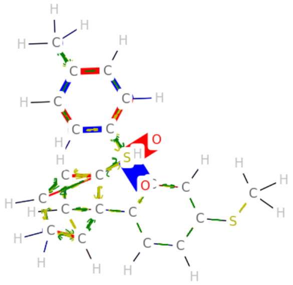

1252	1277	$\nu(CC)_{phenyl}$, $\delta(CH)_{phenyl}$	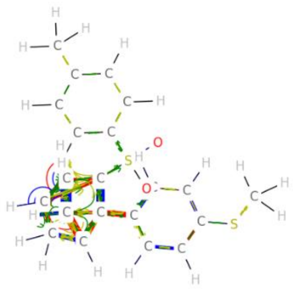
1219	1228	$\nu(CC)_{phenyl}$, $\nu(CO)$, $\delta(CH)_{phenyl}$	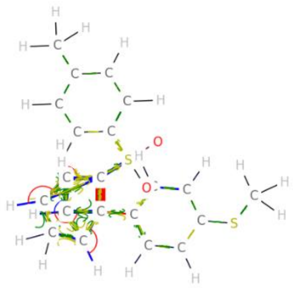
1185	1178	$\nu(CS)$, $\nu(SO)$, $\delta(CH)_{phenyl}$	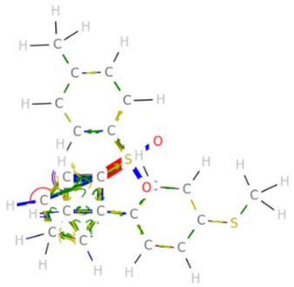
1154	1153	$\nu(CC)$, $\nu(CS)$, $\nu(SO)$	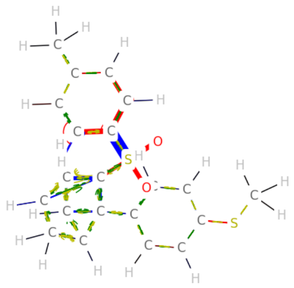

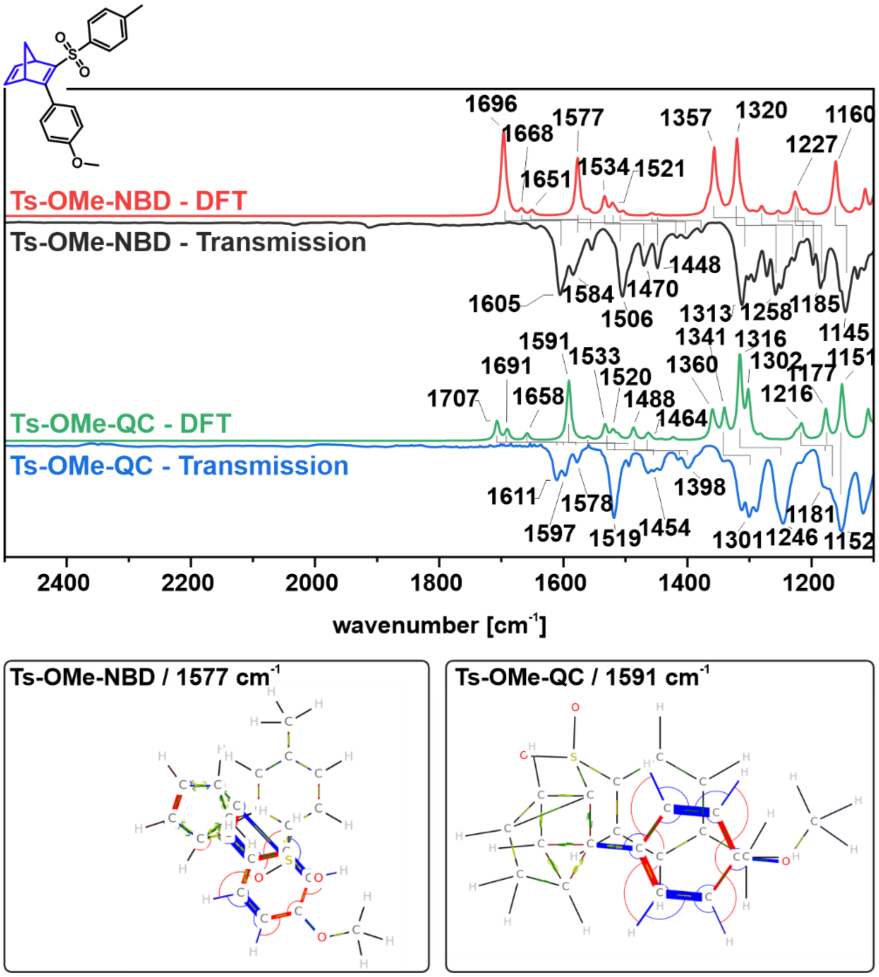

Figure S6: IR spectra of Ts-OMe-NBD. Transmission IR spectra of Ts-OMe-NBD and Ts-OMe-QC and the corresponding spectra calculated by DFT; the spectroscopic marker vibrations $\nu(CC)_{phenyl}$ and $\delta(CH)_{phenyl}$ are visualized by QVibeplot.[11]

Table S11: Band assignment for Ts-OMe-NBD based on transmission spectra and DFT calculations and visualization of the using QVibeplot.[11]

ν_{exp} [cm^{-1}]	ν_{DFT} [cm^{-1}]	vibrational modes	2D representations of the vibrational modes
1605	1696	$\nu(CC)_{phenyl}$, $\nu(CO)$, $\nu(SO)$, $\delta(CH)_{phenyl}$	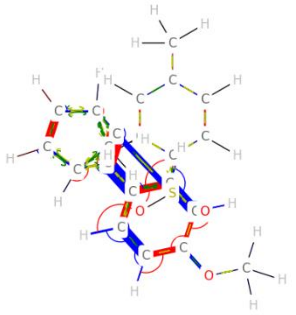
1584	1668	$\nu(CC)_{phenyl}$, $\nu(CS)$, $\delta(CH)_{phenyl}$	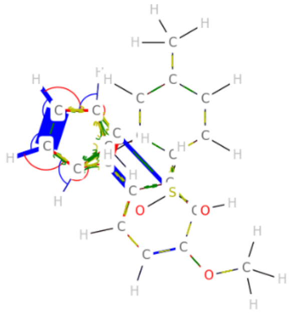
1555	1651	$\nu(CC)_{phenyl}$, $\nu(CS)$, $\delta(CH)_{phenyl}$	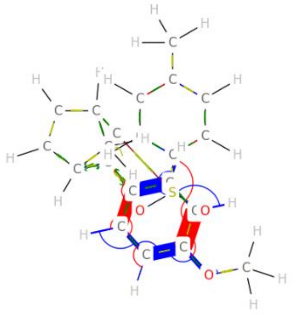
1506	1534	$\delta(CH)_{methyl}$	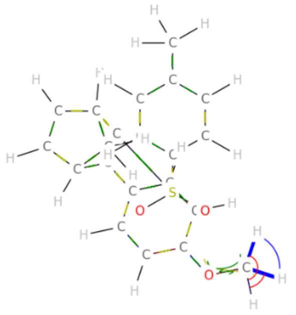

1470	1521	δ(CH)$_{methyl}$	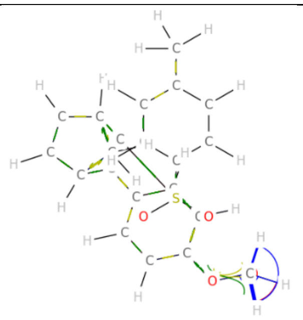
1448	1499	ν(CO), δ(CH)$_{methyl}$	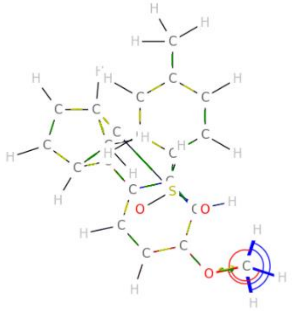
1418	1466	ν(CC)$_{phenyl}$, ν(CS), ν(SO), δ(CH)$_{phenyl}$	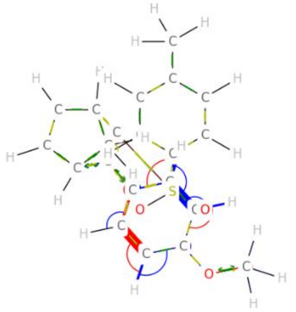
1401	1459	ν(CC)$_{phenyl}$, δ(CH)$_{phenyl}$	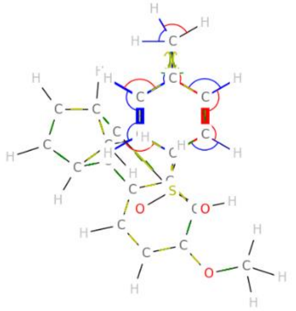
1376	1446	δ(CH)$_{phenyl}$	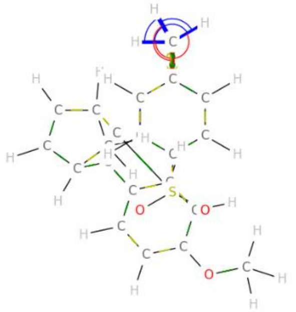

1313	1357	$\nu(CC)_{phenyl}$, $\nu(CS)$, $\nu(CO)$, $\delta(CH)_{phenyl}$	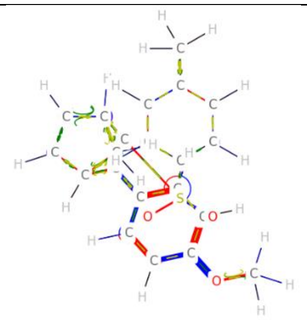
1258	1320	$\nu(CC)_{phenyl}$, $\nu(SO)$, $\nu(CH)$, $\nu(CO)$, $\delta(CH)_{phenyl}$	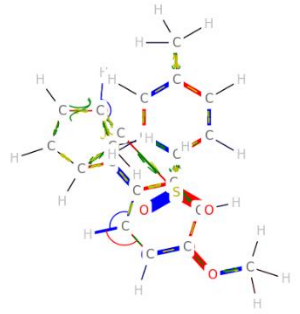
1237	1279	$\nu(CC)_{phenyl}$, $\nu(SO)$, $\nu(CH)$, $\nu(CO)$, $\delta(CH)_{phenyl}$	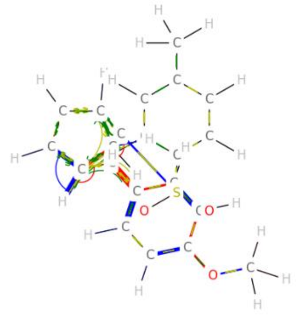
1227	1254	$\nu(CC)_{phenyl}$, $\nu(CH)$, $\delta(CH)_{phenyl}$	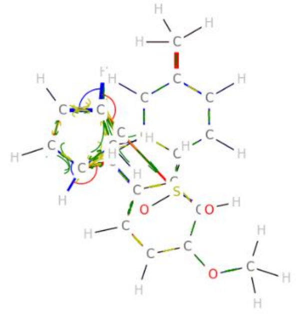
1185	1227	$\nu(CO)$, $\delta(CH)_{methyl}$	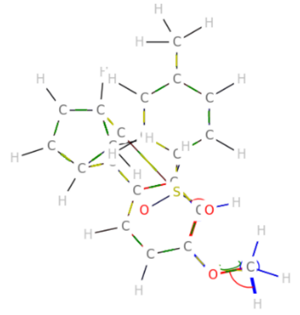

1145	1160	ν(SO), ν(CH)	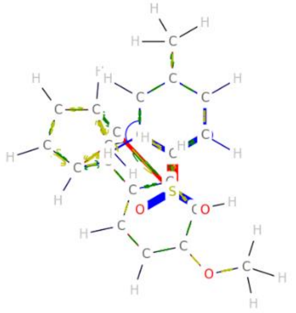

Table S12: Band assignment for Ts-OMe-QC based on transmission spectra and DFT calculations and visualization of the vibrational modes using QVibeplot.[11]

ν_{exp} [cm^{-1}]	ν_{DFT} [cm^{-1}]	vibrational modes	2D representations of the vibrational modes
1611	1707	$\nu(CC)_{phenyl}$, $\delta(CH)_{phenyl}$	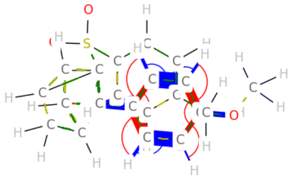
1597	1691	$\nu(CC)_{phenyl}$, $\delta(CH)_{phenyl}$	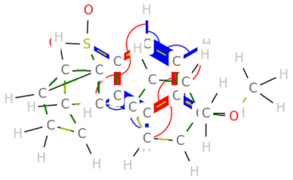
1578	1658	$\nu(CC)_{phenyl}$, $\delta(CH)_{phenyl}$	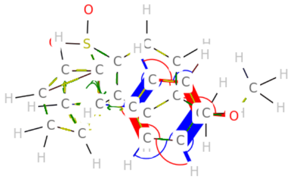

1519	1591	$\nu(CC)_{phenyl}$, $\nu(CO)$, $\delta(CH)_{phenyl}$	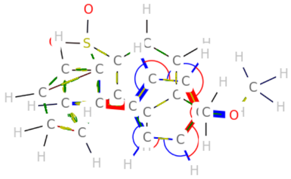
1454	1533	$\delta(CH)_{methyl}$	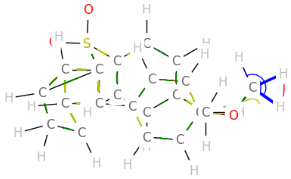
1398	1464	$\nu(CC)_{phenyl}$, $\delta(CH)_{phenyl}$	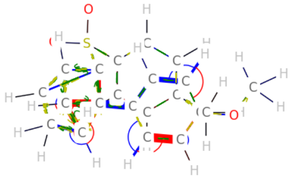
1301	1360	$\nu(CC)_{phenyl}$, $\nu(CO)$, $\delta(CH)_{phenyl}$, $\delta(CH)_{methyl}$	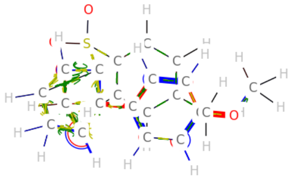
1246	1316	$\nu(SO)$, $\nu(CC)_{phenyl}$, $\nu(CO)$, $\delta(CH)_{methyl}$	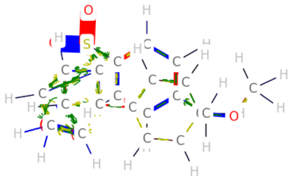

1181	1216	ν(CC)$_{phenyl,}$ δ(CH)$_{methyl}$	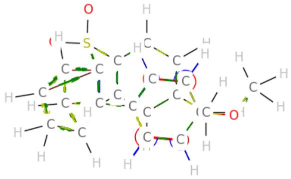
1152	1177	ν(SO), ν(CH), δ(CH)$_{methyl}$	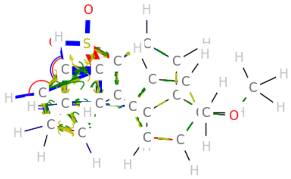

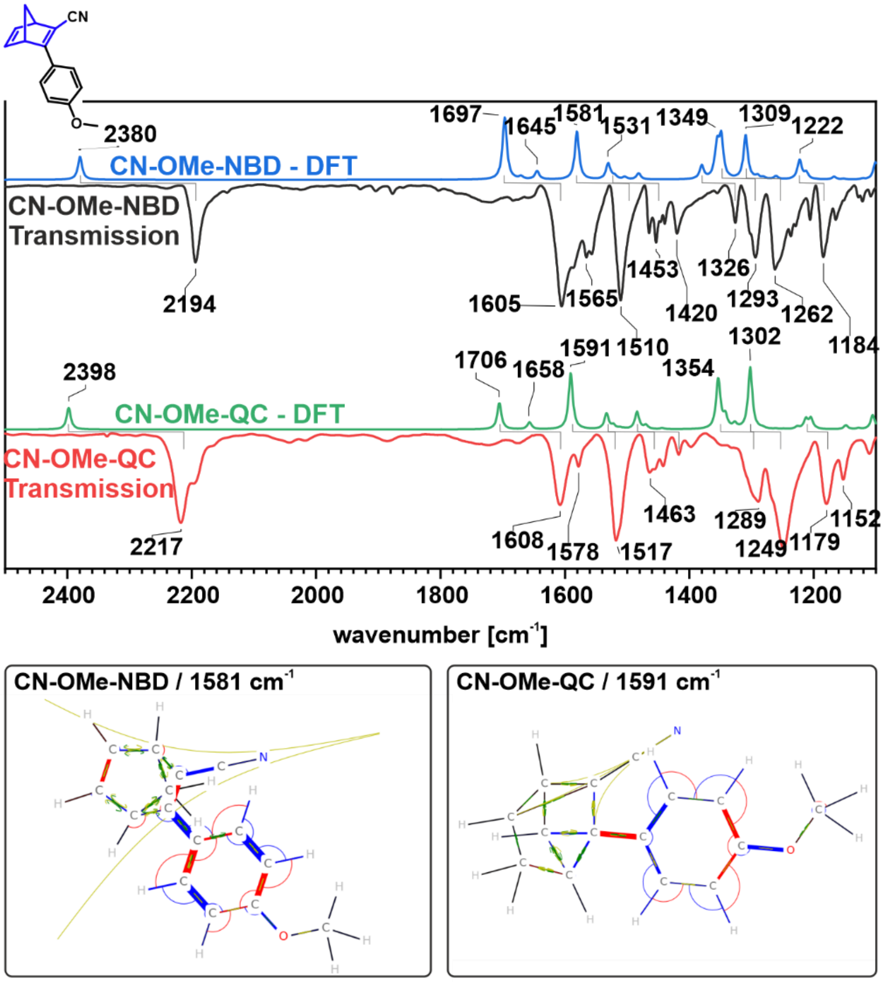

Figure S7: IR spectra of CN-OMe-NBD. Transmission IR spectra of CN-OMe-NBD and CN-OMe-QC and the corresponding spectra calculated by DFT; the spectroscopic marker vibrations $\nu(CC)_{phenyl}$ and $\delta(CH)_{phenyl}$ are visualized by QVibeplot.[11]

Table S13: Band assignment for CN-OMe-NBD based on transmission spectra and DFT calculations and visualization of the vibrational modes using QVibeplot.[11]

ν_{exp} [cm^{-1}]	ν_{DFT} [cm^{-1}]	vibrational modes	2D representations of the vibrational modes
1605	1697	$\nu(CC)_{phenyl}$, $\delta(CH)_{phenyl}$	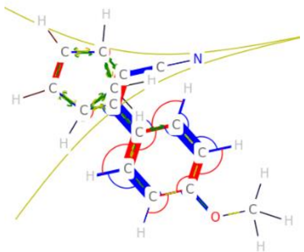
1510	1581	$\nu(CC)_{phenyl}$, $\nu(CO)$, $\delta(CH)_{phenyl}$	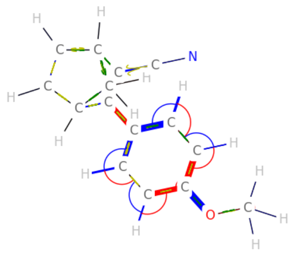
1453	1531	$\delta(CH)_{methyl}$	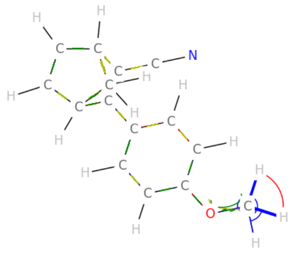
1326	1382	$\nu(CC)_{phenyl}$, $\delta(CH)_{phenyl}$	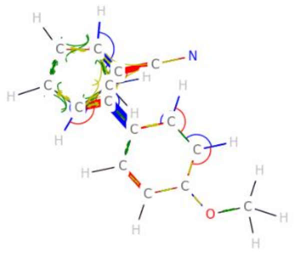

1293	1349	ν(CC)$_{phenyl,}$ ν(CO), δ(CH)$_{phenyl}$	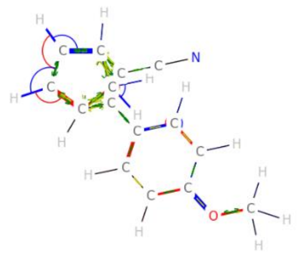
1262	1309	ν(CC)$_{phenyl,}$ ν(CO), δ(CH)$_{phenyl}$	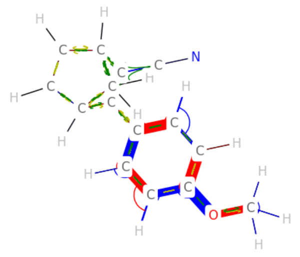
1184	1222	δ(CH)$_{phenyl}$	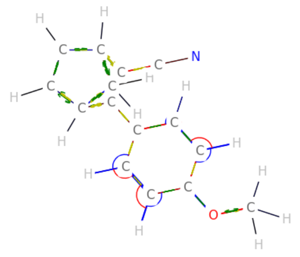

Table S14: Band assignment for CN-OMe-QC based on transmission spectra and DFT calculations and visualization of the using QVibeplot.[11]

ν_{exp} [cm^{-1}]	ν_{DFT} [cm^{-1}]	vibrational modes	2D representations of the vibrational modes
1608	1706	$\nu(CC)_{phenyl,}$ $\nu(CO)$, $\delta(CH)_{phenyl}$	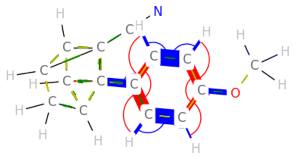
1517	1591	$\nu(CC)_{phenyl,}$ $\nu(CO)$, $\delta(CH)_{phenyl}$	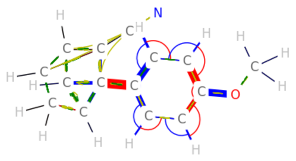
1463	1484	$\nu(CC)_{phenyl,}$ $\delta(CH)_{phenyl}$	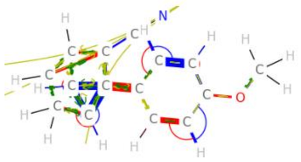
1416	1470	$\nu(CC)_{phenyl,}$ $\delta(CH)_{phenyl}$	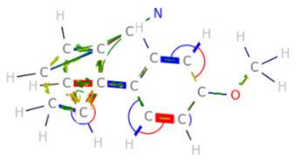

1289	1354	ν(CC)$_{phenyl,}$ ν(CO), δ(CH)$_{phenyl}$	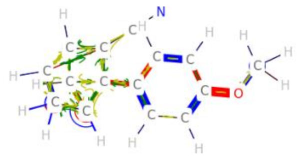
1249	1302	ν(CC)$_{phenyl,}$ ν(CO), δ(CH)$_{phenyl}$	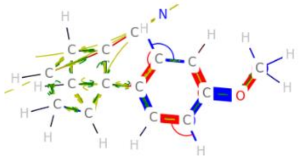
1179	1216	δ(CH)$_{methyl}$	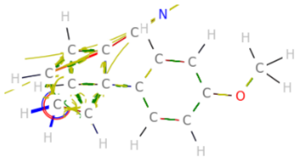
1152	1202	ν(CC)$_{phenyl,}$ δ(CH)$_{phenyl}$	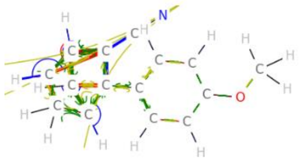

3. Full spectra of the electrochemically triggered back-conversion

In the following, we show the full IR spectra of the electrochemically triggered back-conversion of the investigated systems on HOPG (discussed in detail in the main text). Characteristic bands appear after irradiation. Positive bands appear, which we attribute to the NBD derivative. We also observe negative bands, which we attribute to the QC formed. In the difference spectra shown, positive (up) bands correspond to consumed species, while negative (down) bands correspond to the formation of a species relative to the measured background. Additional features at 1450 cm^{-1}, 2250 cm^{-1}, 2413 cm^{-1}, and 2627 cm^{-1} are associated with the MeCN.[12] We also plotted the concentration as a function of the applied potential for both the main dataset (square markers) and a control experiment to verify reproducibility (round markers). Note that once a potential inducing irreversible decomposition is applied, background instabilities may arise, preventing further quantitative analysis.

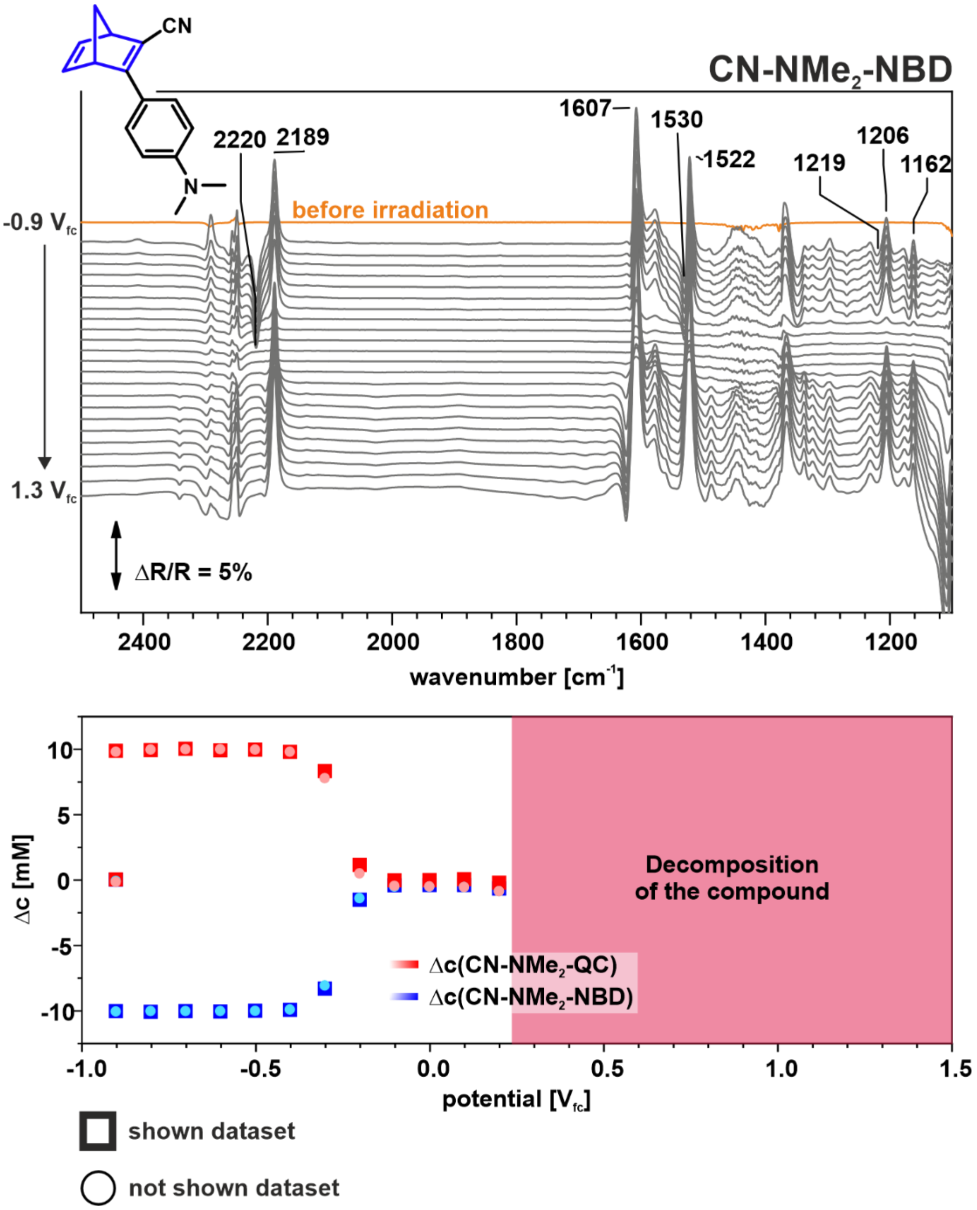

Figure S8: Electrochemically triggered back-conversion of CN-NMe$_2$-NBD. (a) IRRA-spectra of the photochemical conversion and electrochemical back-conversion in the CN-NMe$_2$-NBD/CN-NMe$_2$-QC system on HOPG; (b) change in concentration of CN-NMe$_2$-NBD/CN-NMe$_2$-QC during the electrochemically triggered back-conversion, shown for the main dataset (square markers) and a control experiment to verify reproducibility (round markers).

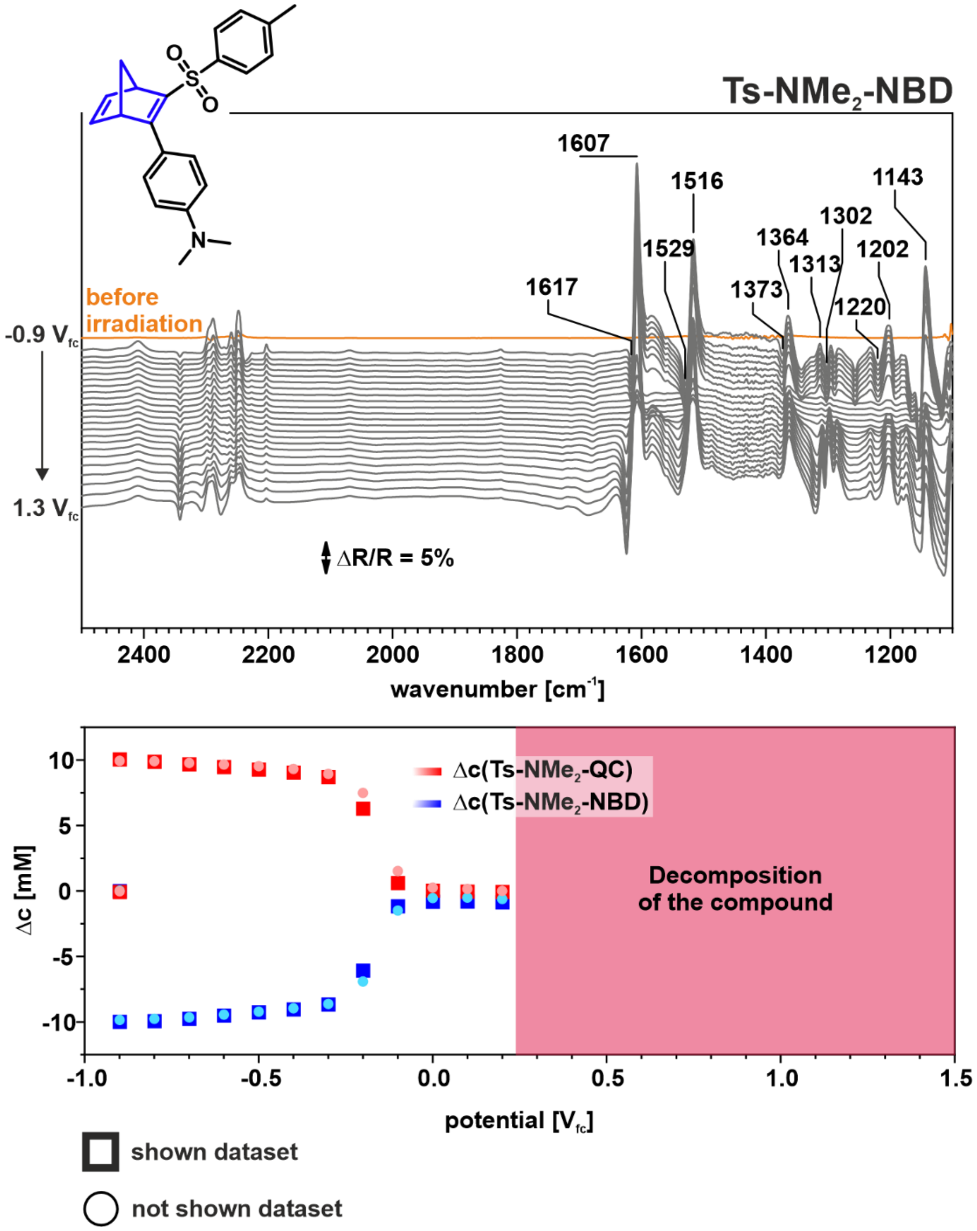

Figure S9: Electrochemically triggered back-conversion of Ts-NMe$_2$-NBD. (a) IRRA-spectra of the photochemical conversion and electrochemical back-conversion in the Ts-NMe$_2$-NBD/Ts-NMe$_2$-QC system on HOPG; (b) change in concentration of Ts-NMe$_2$-NBD/Ts-NMe$_2$-QC during the electrochemically triggered back-conversion, shown for the main dataset (square markers) and a control experiment to verify reproducibility (round markers).

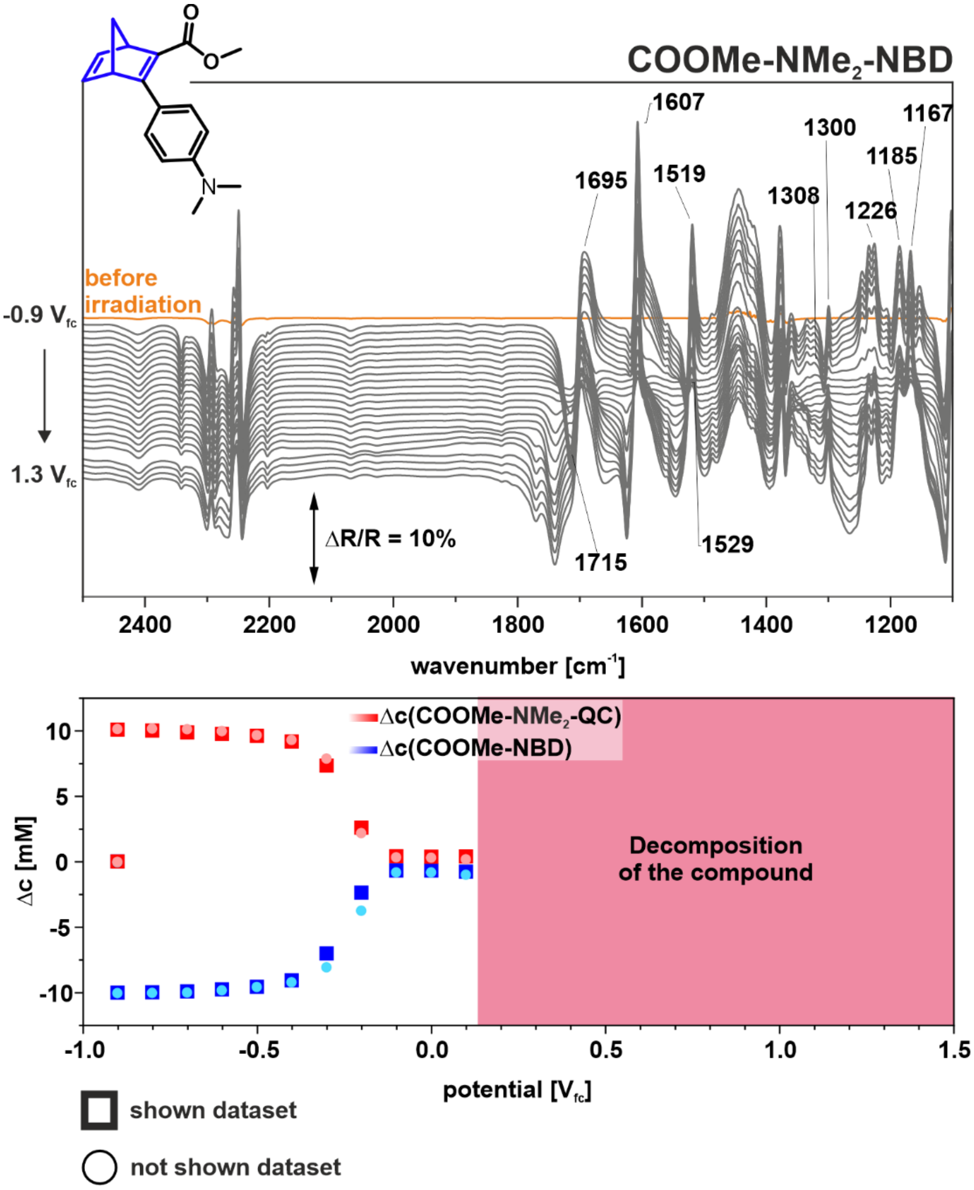

Figure S10: Electrochemically triggered back-conversion of COOMe-NMe$_2$-NBD. (a) IRRA-spectra of the photochemical conversion and electrochemical back-conversion in the COOMe-NMe$_2$-NBD/COOMe-NMe$_2$-QC system on HOPG; (b) change in concentration of COOMe-NMe$_2$-NBD/COOMe-NMe$_2$-QC during the electrochemically triggered back-conversion, shown for the main dataset (square markers) and a control experiment to verify reproducibility (round markers).

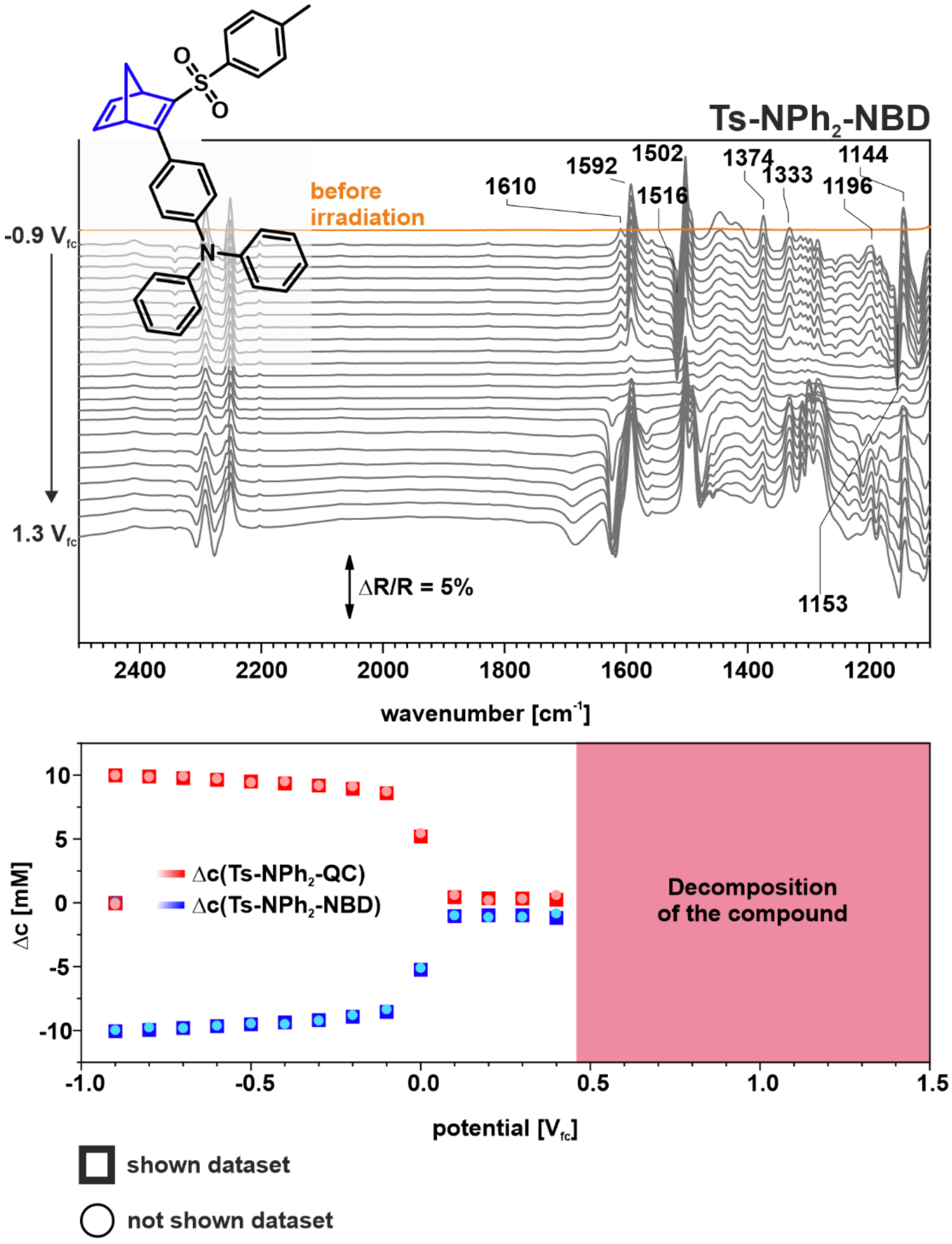

Figure S11: Electrochemically triggered back-conversion of Ts-NPh$_2$-NBD. (a) IRRA-spectra of the photochemical conversion and electrochemical back-conversion in the Ts-NPh$_2$-NBD/Ts-NPh$_2$-QC system on HOPG; (b) change in concentration of Ts-NPh$_2$-NBD/Ts-NPh$_2$-QC during the electrochemically triggered back-conversion, shown for the main dataset (square markers) and a control experiment to verify reproducibility (round markers).

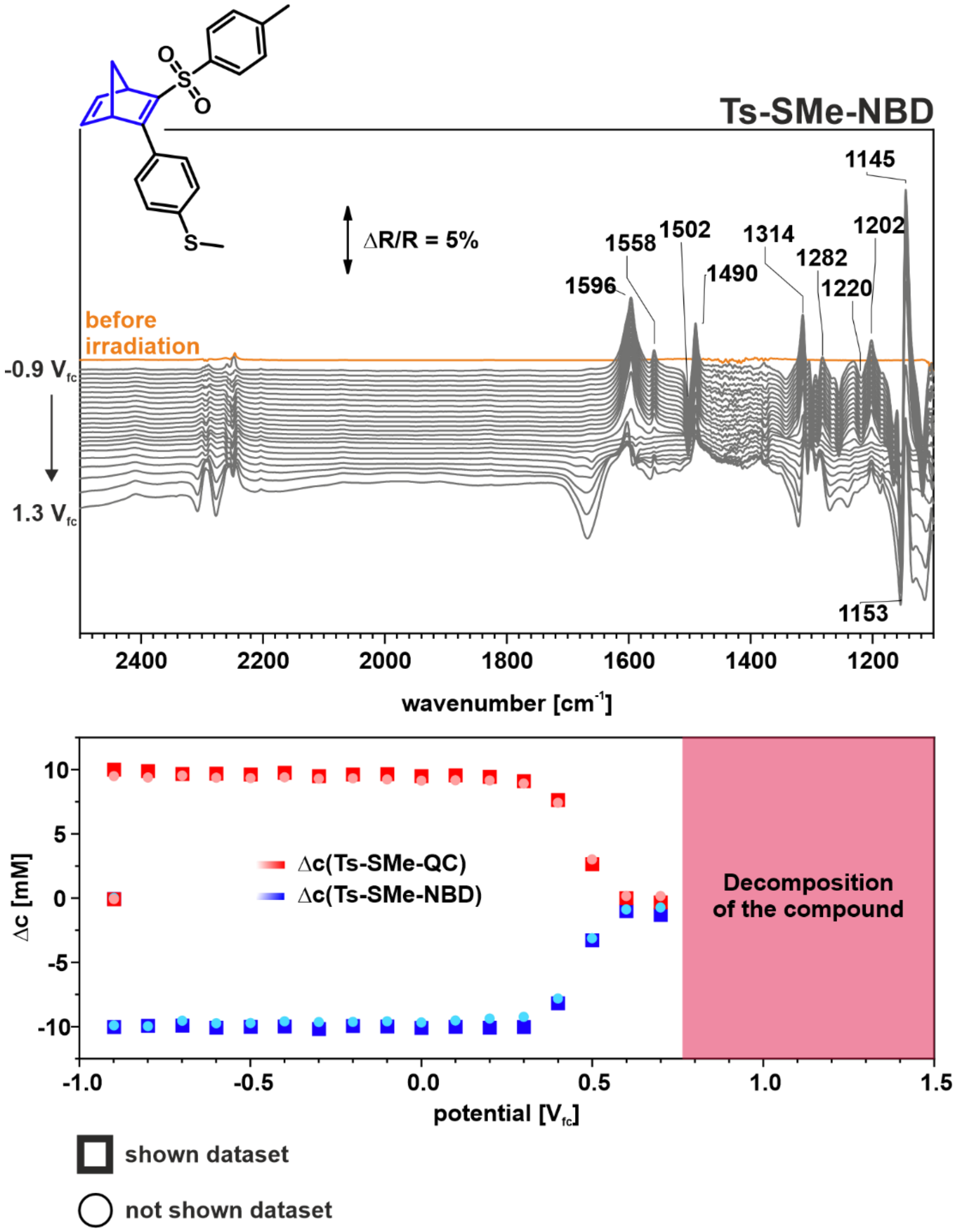

Figure S12: Electrochemically triggered back-conversion of Ts-SMe-NBD. (a) IRRA-spectra of the photochemical conversion and electrochemical back-conversion in the Ts-SMe-NBD/Ts-SMe-QC system on HOPG; (b) change in concentration of Ts-SMe-NBD/Ts-SMe-QC during the electrochemically triggered back-conversion, shown for the main dataset (square markers) and a control experiment to verify reproducibility (round markers).

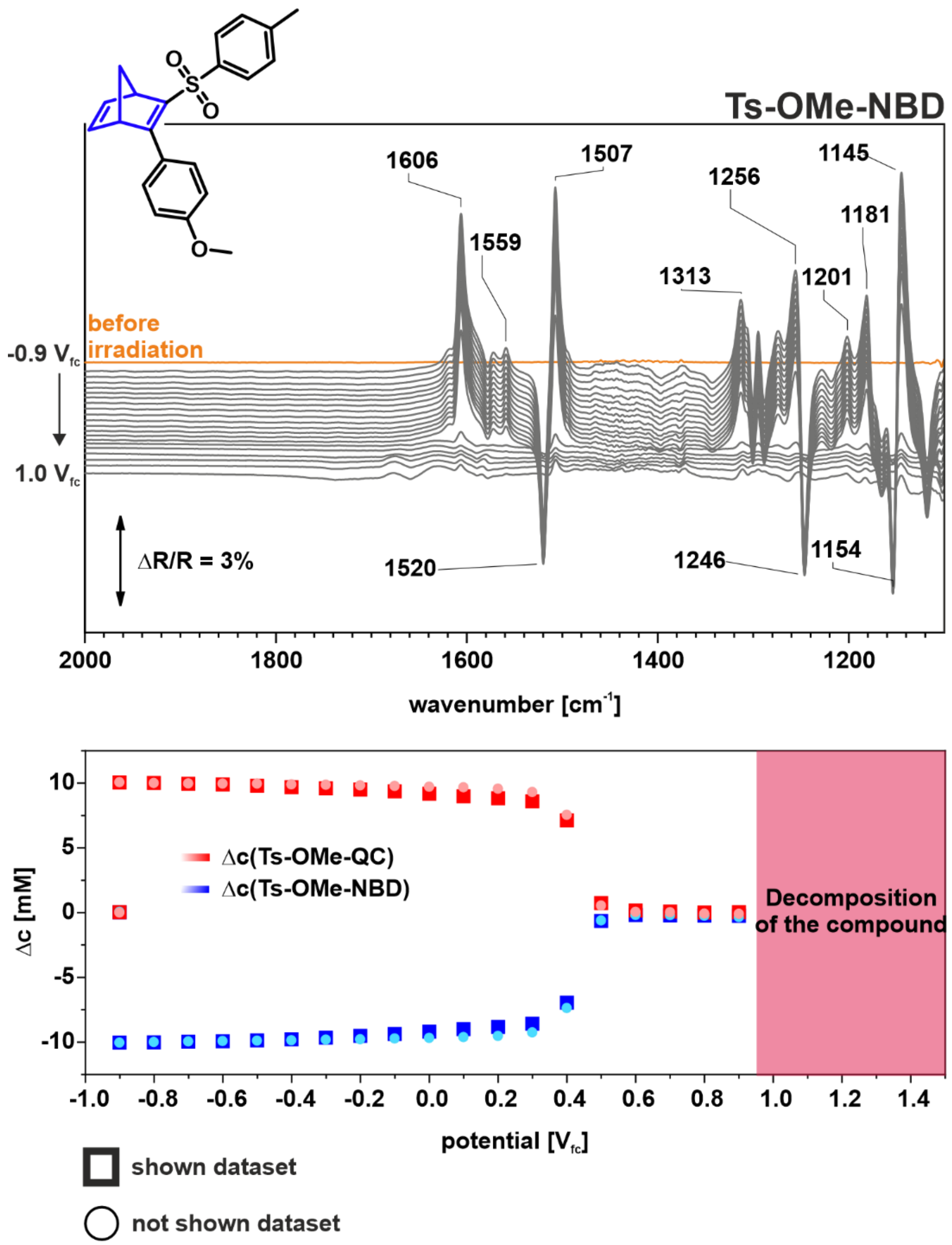

Figure S13: Electrochemically triggered back-conversion of Ts-OMe-NBD. (a) IRRA-spectra of the photochemical conversion and electrochemical back-conversion in the Ts-OMe-NBD/Ts-OMe-QC system on HOPG; (b) change in concentration of Ts-OMe-NBD/Ts-OMe-QC during the electrochemically triggered back-conversion, shown for the main dataset (square markers) and a control experiment to verify reproducibility (round markers).

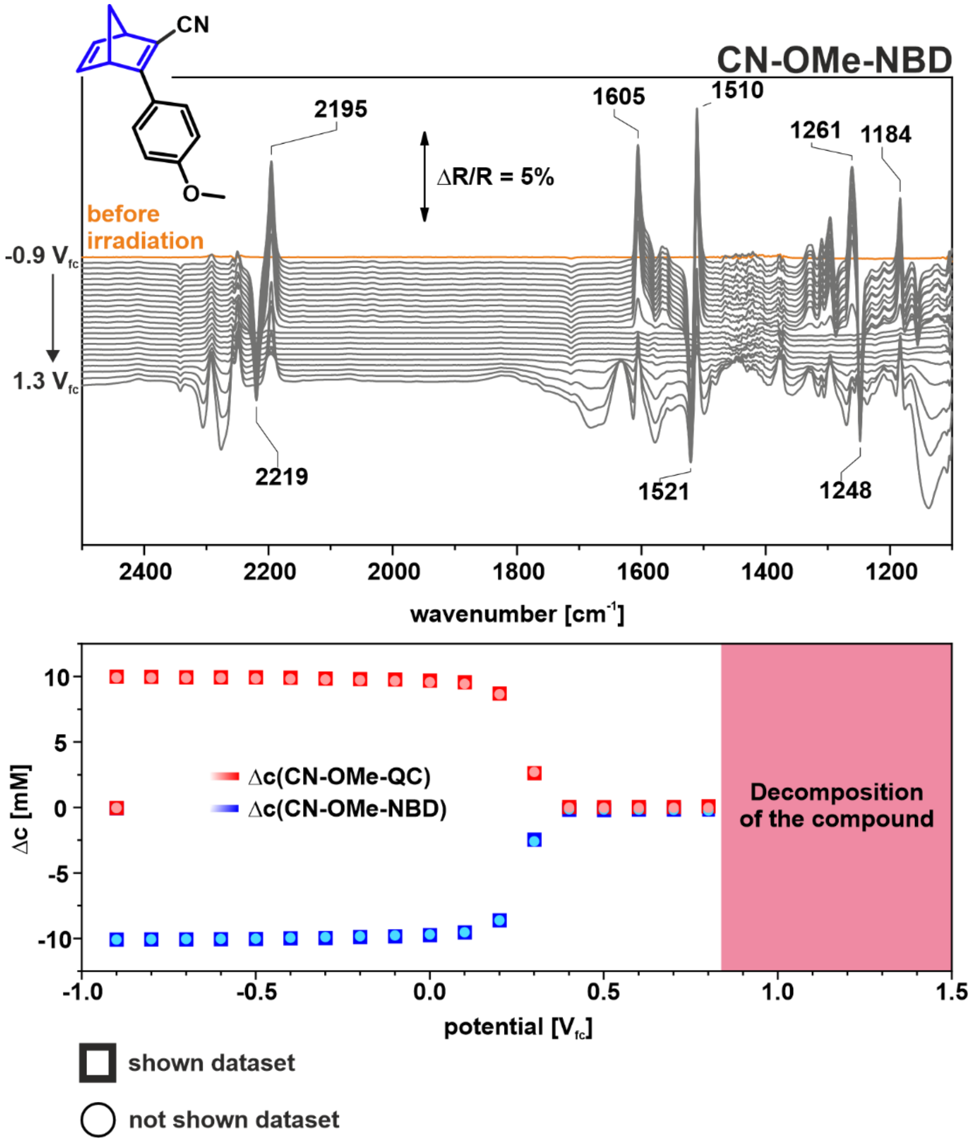

Figure S14: Electrochemically triggered back-conversion of CN-OMe-NBD. (a) IRRA-spectra of the photochemical conversion and electrochemical back-conversion in the CN-OMe-NBD/CN-OMe-QC system on HOPG; (b) change in concentration of CN-OMe-NBD/CN-OMe-QC during the electrochemically triggered back-conversion, shown for the main dataset (square markers) and a control experiment to verify reproducibility (round markers).

4. Calculated Energies of the HOMO and LUMO

Compound	Energy of HOMO [eV]	Energy of the LUMO [eV]	HOMO-LUMO gap [eV]
**CN-NMe$_2$-QC**	-6.62325476	1.12383082	7.74708558
**Ts-NMe$_2$-QC**	-6.7076101	0.31565224	7.02326234
**COOMe-NMe$_2$-QC**	-6.48175548	1.33063746	7.81239294
**Ts-NPh$_2$-QC**	-6.54978398	0.16054726	6.71033124
**Ts-SMe-QC**	-7.8777003	0.03537482	7.91307512
**Ts-OMe-QC**	-7.42054878	0.19592208	7.61647086
**CN-OMe-QC**	-7.41782764	0.93062988	8.34845752

Compound	Energy of HOMO [eV]	Energy of the LUMO [eV]	HOMO-LUMO gap [eV]
**CN-NMe$_2$-NBD**	-6.6395816	-0.47347836	6.16610324
**Ts-NMe$_2$-NBD**	-6.60420678	-0.38368074	6.22052604
**COOMe-NMe$_2$-NBD**	-6.52529372	-0.0952399	6.43005382
**Ts-NPh$_2$-NBD**	-6.60148564	-0.39728644	6.2041992
**Ts-SMe-NBD**	-7.74436444	-0.57416054	7.1702039
**Ts-OMe-NBD**	-7.24639582	-0.48436292	6.7620329
**CN-OMe-NBD**	-7.2246267	-0.53334344	6.69128326

5. Calculated Energies of the Compounds

Compound	Energy of the NBD [hartree]	Energy of the NBD^+ [hartree]	Energy of the QC [hartree]	Energy of the QC^+ [hartree]
**CN-NMe$_2$-NBD**	-728.53844	-728.28838	-728.49624	-728.25171
**Ts-NMe$_2$-NBD**	-1455.179	-1454.9374	-1455.1547	-1454.9075
**COOMe-NMe$_2$-NBD**	-864.16067	-863.91567	-864.12572	-863.88587
**Ts-NPh$_2$-NBD**	-1838.5503	-1838.3023	-1838.5195	-1838.2758
**Ts-SMe-NBD**	-1758.755	-1758.491	-1758.7319	-1758.4626
**Ts-OMe-NBD**	-1435.7568	-1435.4933	-1435.7326	-1435.4662
**CN-OMe-NBD**	-709.11454	-708.84337	-709.0749	-708.80783

6. Onset Potential Analysis

The onset potentials for both the back-conversion and decomposition processes can be determined directly from the spectra (see Figure S15) as well as from the resulting quantitative analysis (see Figure 2d, main manuscript). The onset potential for the back-conversion is identified in the spectra as the point at which the initially stable bands (both NBD and QC) begin to decrease significantly following the photochemical transformation (see red spectrum in Figure S15a). This corresponds, in the quantitative analysis, to a decrease in QC concentration and a simultaneous increase in NBD concentration (see –0.2 V_{fc} in Figure 2d).

The onset potential for the oxidative decomposition of NBD is identified as the point at which the positive NBD bands begin to increase again after the electrochemically triggered back-conversion (see red spectrum in Figure S15b). In the quantitative analysis, this is reflected by a decrease in NBD concentration (see 0.3 V_{fc} in Figure 2d).

Due to general spectral background instability caused by decomposition, accurate quantitative analysis beyond this point becomes difficult in some cases and is therefore not shown in Figures S8–S14.

The onset potentials were manually determined based on both the spectral data and the quantitative analysis.

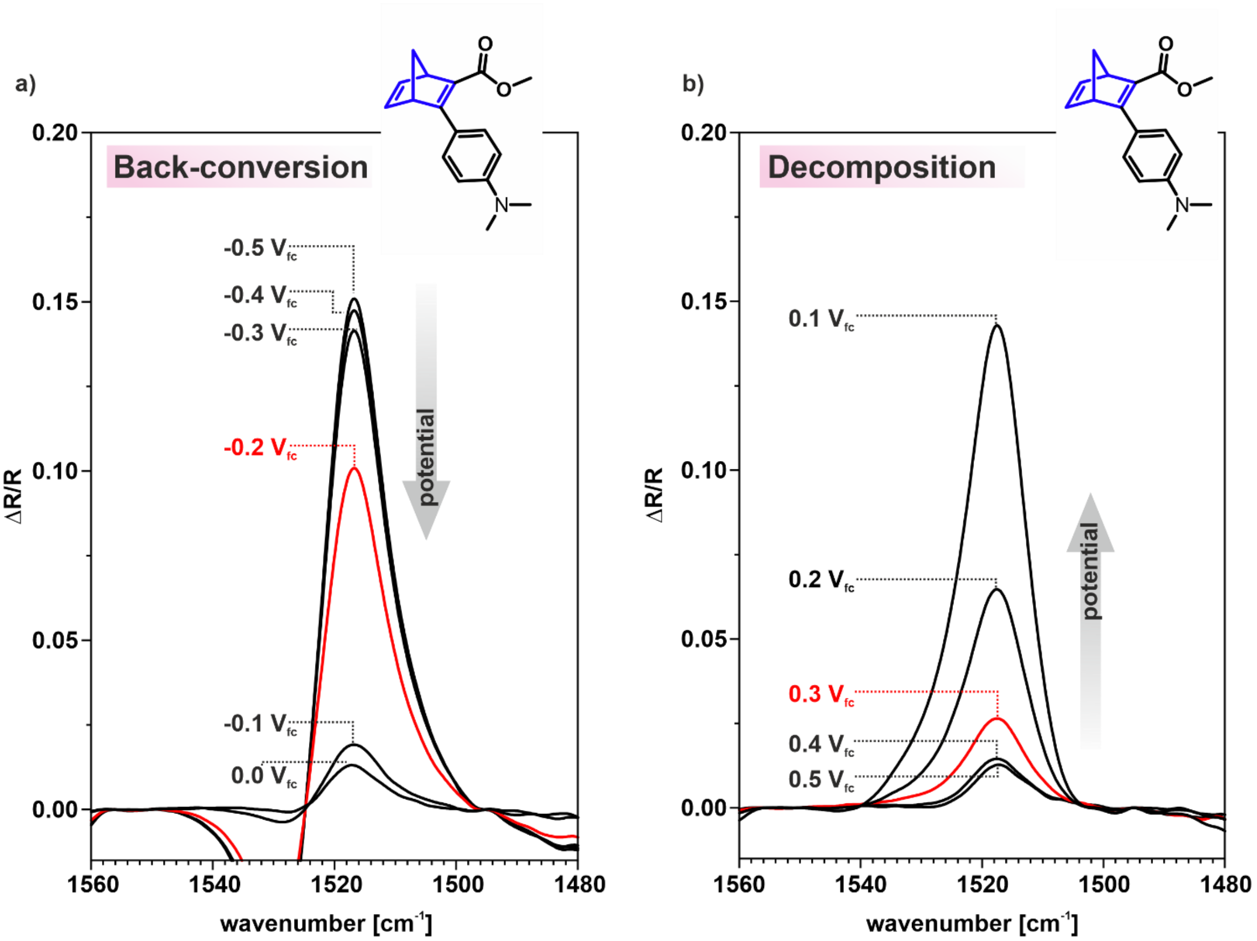

Figure S15: IRRA spectra of the Ts-NMe_2-NBD/QC system recorded between –0.5 and 0 V_{fc} (a) and between 0.1 and 0.5 V_{fc} (b), illustrating the electrochemically triggered back-conversion (a) and oxidative decomposition (b), respectively. The red spectra indicate the onset potentials for the respective processes.

7. References

[1] Y. Shao, Z. Gan, E. Epifanovsky, A. T. B. Gilbert, M. Wormit, J. Kussmann, A. W. Lange, A. Behn, J. Deng, X. Feng, D. Ghosh, M. Goldey, P. R. Horn, L. D. Jacobson, I. Kaliman, R. Z. Khaliullin, T. Kuś, A. Landau, J. Liu, E. I. Proynov, Y. M. Rhee, R. M. Richard, M. A. Rohrdanz, R. P. Steele, E. J. Sundstrom, H. L. Woodcock, P. M. Zimmerman, D. Zuev, B. Albrecht, E. Alguire, B. Austin, G. J. O. Beran, Y. A. Bernard, E. Berquist, K. Brandhorst, K. B. Bravaya, S. T. Brown, D. Casanova, C. M. Chang, Y. Chen, S. H. Chien, K. D. Closser, D. L. Crittenden, M. Diedenhofen, R. A. Distasio, H. Do, A. D. Dutoi, R. G. Edgar, S. Fatehi, L. Fusti-Molnar, A. Ghysels, A. Golubeva-Zadorozhnaya, J. Gomes, M. W. D. Hanson-Heine, P. H. P. Harbach, A. W. Hauser, E. G. Hohenstein, Z. C. Holden, T. C. Jagau, H. Ji, B. Kaduk, K. Khistyaev, J. Kim, J. Kim, R. A. King, P. Klunzinger, D. Kosenkov, T. Kowalczyk, C. M. Krauter, K. U. Lao, A. D. Laurent, K. V. Lawler, S. V. Levchenko, C. Y. Lin, F. Liu, E. Livshits, R. C. Lochan, A. Luenser, P. Manohar, S. F. Manzer, S. P. Mao, N. Mardirossian, A. V. Marenich, S. A. Maurer, N. J. Mayhall, E. Neuscamman, C. M. Oana, R. Olivares-Amaya, D. P. Oneill, J. A. Parkhill, T. M. Perrine, R. Peverati, A. Prociuk, D. R. Rehn, E. Rosta, N. J. Russ, S. M. Sharada, S. Sharma, D. W. Small, A. Sodt, T. Stein, D. Stück, Y. C. Su, A. J. W. Thom, T. Tsuchimochi, V. Vanovschi, L. Vogt, O. Vydrov, T. Wang, M. A. Watson, J. Wenzel, A. White, C. F. Williams, J. Yang, S. Yeganeh, S. R. Yost, Z. Q. You, I. Y. Zhang, X. Zhang, Y. Zhao, B. R. Brooks, G. K. L. Chan, D. M. Chipman, C. J. Cramer, W. A. Goddard, M. S. Gordon, W. J. Hehre, A. Klamt, H. F. Schaefer, M. W. Schmidt, C. D. Sherrill, D. G. Truhlar, A. Warshel, X. Xu, A. Aspuru-Guzik, R. Baer, A. T. Bell, N. A. Besley, J. Da Chai, A. Dreuw, B. D. Dunietz, T. R. Furlani, S. R. Gwaltney, C. P. Hsu, Y. Jung, J. Kong, D. S. Lambrecht, W. Liang, C. Ochsenfeld, V. A. Rassolov, L. V. Slipchenko, J. E. Subotnik, T. Van Voorhis, J. M. Herbert, A. I. Krylov, P. M. W. Gill, M. Head-Gordon, *Mol. Phys.* **2015**, *113*, 184–215.

[2] T. Yanai, D. P. Tew, N. C. Handy, *Chem. Phys. Lett.* **2004**, *393*, 51–57.

[3] R. Krishnan, J. S. Binkley, R. Seeger, J. A. Pople, *J. Chem. Phys.* **1980**, *72*, 650–654.

[4] S. Grimme, J. Antony, S. Ehrlich, H. Krieg, *J. Chem. Phys.* **2010**, *132*, DOI 10.1063/1.3382344.

[5] F. Coppola, M. Nucci, M. Marazzi, D. Rocca, M. Pastore, *ChemPhotoChem* **2023**, *7*, e202200214.

[6] J. C. Cooper, A. Kirrander, *Phys. Chem. Chem. Phys.* **2025**, *27*, 3089–3101.

[7] M. Jevric, A. U. Petersen, M. Mansø, S. Kumar Singh, Z. Wang, A. Dreos, C. Sumby, M. B. Nielsen, K. Börjesson, P. Erhart, K. Moth-Poulsen, *Chem. - A Eur. J.* **2018**, *24*, 12767–12772.

[8] J. Orrego-Hernández, H. Hölzel, M. Quant, Z. Wang, K. Moth-Poulsen, *European J. Org. Chem.* **2021**, *2021*, 5337–5342.

[9] D. Krappmann, A. Hirsch, *Chem. – A Eur. J.* **2024**, DOI 10.1002/chem.202401391.

[10] P. Lorenz, T. Luchs, A. Hirsch, *Chem. – A Eur. J.* **2021**, *27*, 4993–5002.

[11] M. Laurin, *J. Chem. Educ.* **2013**, *90*, 944–946.

[12] E. L. Pace, L. J. Noe, *J. Chem. Phys.* **1968**, *49*, 5317–5325.
